# Autophagy Reprograms Alveolar Progenitor Cell Metabolism in Response to Lung Injury

**DOI:** 10.1016/j.stemcr.2020.01.008

**Published:** 2020-02-13

**Authors:** Xue Li, Junping Wu, Xin Sun, Qi Wu, Yue Li, Kuan Li, Qiuyang Zhang, Yu Li, E. Dale Abel, Huaiyong Chen

**Affiliations:** 1 Department of Basic Medicine, Tianjin University Haihe Hospital, Tianjin 300350, China; 2 Tianjin Key Laboratory of Lung Regenerative Medicine, Tianjin, China; 3 Key Research Laboratory for Infectious Disease Prevention for State Administration of Traditional Chinese Medicine, Tianjin Institute of Respiratory Diseases, Tianjin, China; 4 Department of Basic Medicine, Haihe Clinical College of Tianjin Medical University, Tianjin, China; 5 Fraternal Order of Eagles Diabetes Research Center and Division of Endocrinology and Metabolism, Roy J. and Lucille A. Carver College of Medicine, University of Iowa, Iowa City, IA, USA

**Keywords:** autophagy, AT2 progenitor cells, lung injury, glucose metabolism, lipid metabolism, oxidative stress, metabolic reprogramming, organoids

## Abstract

Autophagy is a protective cellular mechanism in response to stress conditions. However, whether autophagy is required for maintenance of the alveolar epithelium is unknown. Here, we report that the loss of autophagy-related 5 (Atg5) in AT2 cells worsened bleomycin-induced lung injury. Mechanistically, during bleomycin injury, autophagy downregulated lipid metabolism but upregulated glucose metabolism in AT2 cells for alveolar repair. Chemical blockade of fatty acid synthesis promoted organoid growth of AT2 cells and counteracted the effects of autophagy loss on bleomycin injury. However, genetic loss of glucose transporter 1, interference with glycolysis, or interference with the pentose phosphate pathway reduced the proliferation of AT2 cells. Inhibition of glucose metabolism exacerbated the effects of bleomycin injury. Failure of autophagy generated additional hydrogen peroxide, which reduced AT2 cell proliferation. These data highlight an essential role for autophagy in reprogramming the metabolism of alveolar progenitor cells to meet energy needs for alveolar epithelial regeneration.

## Highlights

• Defective autophagy in AT2 cells worsens bleomycin-induced lung injury
• Autophagy switches lipid metabolism to glucose metabolism in AT2 cells for repair
• Chemical blockade of fatty acid synthesis promotes organoid growth of AT2 cells
• Glucose metabolism favors AT2 cell proliferation and limits bleomycin injury

## Teaser

Metabolic reprogramming in stem/progenitor cells is essential for tissue maintenance and regeneration after injuries. In this article, Chen and colleagues report that autophagy protects from bleomycin-induced lung injury by switching lipid metabolism to glucose metabolism in AT2 progenitor cells. Repairing capacity of AT2 cells is positively regulated by glucose metabolism, but negatively controlled by lipid metabolism.

## Introduction

Tissue maintenance and successful regeneration after injury rely on healthy stem/progenitor cells. Dysfunctional stem/progenitor cells will limit regeneration, increasing vulnerability to insults at steady state or persistence of tissue injury. The induction of senescence by oxidative stress contributes to stem cell impairment in a variety of tissues (Baraibar et al., 2016, Iglesias-Bartolome et al., 2012, Moorefield et al., 2017). During bleomycin-induced injury in the adult murine lung, senescence markers are upregulated in alveolar type 2 (AT2) cells, which self-renew and differentiate into AT1 cells as stem/progenitor cells (Lehmann et al., 2017). However, it remains unclear how AT2 cells maintain their health during homeostasis and injury repair.

Accumulating evidence suggests that the metabolic program of stem cells is critical for their maintenance through influencing the balance between self-renewal and differentiation. In the intestinal epithelium, glycolysis in niche Paneth cells produces lactate, which is converted into pyruvate by lactate dehydrogenase in Lgr5^+^ intestinal stem cells to support organoid formation (Rodriguez-Colman et al., 2017). The blockade of pyruvate import into mitochondria for further oxidation promotes intestinal stem cell maintenance and proliferation (Schell et al., 2017). Lactate dehydrogenase activity increases during hair follicle stem cell activation, while deletion of this catalytic enzyme blocks hair follicle stem cell activation and the hair cell cycle (Flores et al., 2017).

In addition to glucose metabolism, lipid metabolism affects stem cell function. Fasting augments intestinal stem cell function by inducing fatty acid oxidation (Mihaylova et al., 2018). Deletion of fatty acid synthase impairs the proliferation of neural stem and progenitor cells (Knobloch et al., 2013). Therefore, an understanding of how these metabolic programs are regulated in stem/progenitor cells is essential for the clinical restoration of tissue homeostasis after injury through targeting of the regeneration machinery.

Autophagy is induced during nutrient and energy deprivation in many cells as a homeostatic response to metabolic stress. Cytosolic components are engulfed and sealed into autophagosomes in a process directed by autophagy-related (Atgs) genes. Autophagosome contents are then delivered to lysosomes for digestion to reutilize for energy (Singh and Cuervo, 2011). Loss-of-function studies have demonstrated that autophagy also maintains homeostasis in a number of tissues, including intestinal, liver, and adipose, by regulating the proliferation and differentiation of local stem/progenitor cells or by preventing senescence or necroptosis at steady state and after injury repair (Garcia-Prat et al., 2016, Matsuzawa-Ishimoto et al., 2017, Singh et al., 2009, Zeng et al., 2016). Alveolar injury induced by bleomycin has been found to be associated with compromised autophagy in a mouse model (Gui et al., 2015). Induction of autophagy protects epithelial cells against bleomycin-induced stress and apoptosis (Cabrera et al., 2015, Divya et al., 2017, Gui et al., 2015). However, how alveolar regeneration is influenced by autophagy and metabolic states after injury is unclear.

In this study, we used RNA sequencing (RNA-seq), metabolomics, and genetic loss-of-function analyses to demonstrate that bleomycin-induced alveolar injury alters glucose and lipid metabolism, which is regulated by autophagy. Observations from flow cytometry, immunofluorescence, and organoid cultures indicated the differential role of autophagy in regulating AT2 function in both steady state and injury conditions. Genetic knockdown of glucose transporter 1 (*Glut1*), selective inhibition of metabolic enzymes in glycolysis, or inhibition of the pentose phosphate pathway impaired the proliferation of mouse AT2 cells. Unlike glucose metabolism, blocking fatty acid synthesis promoted proliferation. Furthermore, hydrogen peroxide dampened AT2 cell proliferation. The results provide evidence of the essential role of autophagy in driving the metabolic switch in alveolar progenitor cells to meet energy needs for lung homeostasis and regeneration.

## Results

### Autophagy Maintains Alveolar Progenitor Cell Pool during BLM-Induced Lung Injury

**Figure 1:**
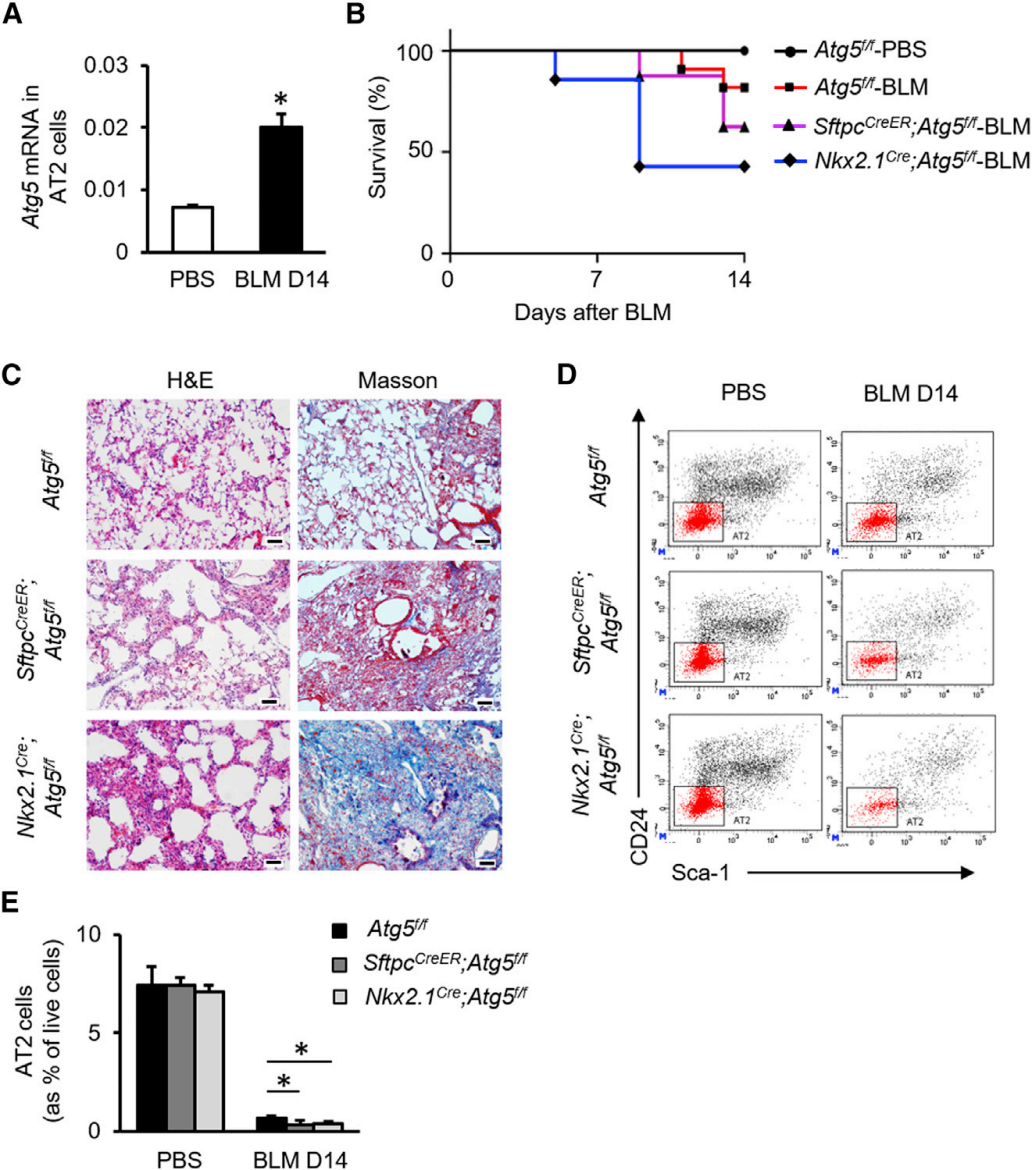
Autophagy Maintains AT2 Cell Pool during BLM-Induced Lung Injury (A) *Atg5* gene expression in mouse AT2 cells after BLM injury (n = 6). (B) Survival of *Atg5*^*f/f*^, *Sftpc*^*CreER*^*;Atg5*^*f/f*^ (pretreated with tamoxifen), or *Nkx2*.*1*^*Cre*^*;Atg5*^*f/f*^ mice after intratracheal instillation of BLM (n = 10). (C) Hematoxylin/eosin staining (left column) and Masson trichrome (right column) staining of lung sections from *Atg5*^*f/f*^, *Sftpc*^*CreER*^*;Atg*^*5f/f*^ (pretreated with tamoxifen), and *Nkx2*.*1*^*Cre*^*;Atg5*^*f/f*^ mice after BLM injury. Scale bar: 50 μm. (D and E) Representative charts of flow cytometric analysis (D) and summarized abundance (E) of survived AT2 cells in lungs of *Atg5*^*f/f*^, *Sftpc*^*CreER*^*;Atg5*^*f/f*^ (pretreated with tamoxifen), or *Nkx2*.*1*^*Cre*^*;Atg5*^*f/f*^ mice after BLM injury (n = 4). Data are representative of two or more independent experiments with error bars representing the mean ± SD. ^∗^p < 0.05.

Autophagy is induced by tissue injury (Hou et al., 2013). We observed that the LC3II/LC3I ratio, a hallmark of autophagy, was significantly increased in mouse lung tissue at 14 days after bleomycin (BLM) administration (Figure S1A). *Atg5* mRNA expression was promoted in the surviving AT2 cells, identified as CD31^−^CD34^−^CD45^−^Sca-1^−^EpCAM^+^CD24^−^ by fluorescence-activated cell sorting (FACS) as described previously (Chen et al., 2012), from *Atg5*^*f/f*^ mice 14 days after BLM administration (Figure 1A). To investigate whether epithelial autophagy is involved in alveolar injury and repair, *Sftpc*^*CreER*^*;Atg5*^*f/f*^ and *Nkx2*.*1*^*Cre*^*;Atg5*^*f/f*^ mice were established to eliminate *Atg5* expression in AT2 cells. Relative to *Atg5*^*f/f*^ mice, *Sftpc*^*CreER*^*;Atg5*^*f/f*^ mice were more susceptible to BLM-induced lung injury (Figure 1B). Airways and alveoli of *Nkx2*.*1*^*Cre*^*;Atg5*^*f/f*^ mice both developed normally, with no readily observable gross or histological abnormalities (Figures S1B–S1J). The survival of *Nkx2*.*1*^*Cre*^*;Atg5*^*f/f*^ mice was further decreased during BLM-induced lung injury (Figure 1B). Relative to *Atg5*^*f/f*^ mice, *Sftpc*^*CreER*^*;Atg5*^*f/f*^ and *Nkx2*.*1*^*Cre*^*;Atg5*^*f/f*^ mice had increased fibrosis at 14 days after BLM challenge, as illustrated by distorted alveolar structure and enhanced trichrome staining (Figure 1C). Flow cytometry indicated a reduction in the proportion of surviving AT2 cells in *Sftpc*^*CreER*^*;Atg5*^*f/f*^ or *Nkx2*.*1*^*Cre*^*;Atg5*^*f/f*^ mice at day 14 relative to *Atg5*^*f/f*^ mice (Figures 1D and 1E). Similar to *Pseudomonas aeruginosa*-induced lung injury (Liu et al., 2015), an Sca-1^+^ alveolar population was observed during BLM-induced lung injury, but there was no difference in colony-forming efficiencies (CFEs) of AT2 cells versus Sca-1^+^ alveolar population (Figure S2).These data suggested that autophagy maintains the AT2 cell pool during BLM-induced lung injury.

### Autophagy Sustains Proliferation of AT2 Cells during BLM Injury

**Figure 2:**
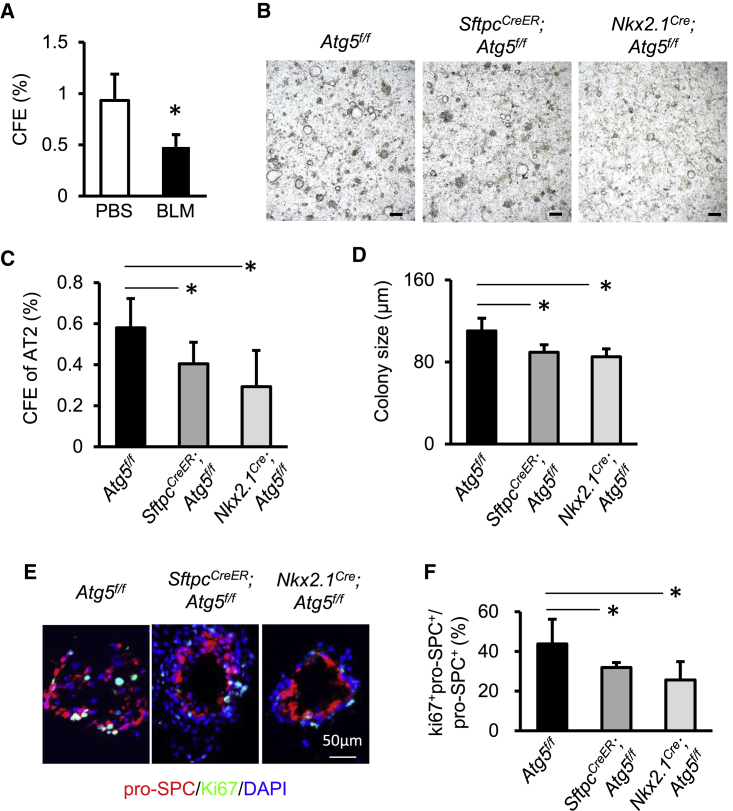
Autophagy Sustains the Proliferative Capacity of AT2 Cells during BLM Injury (A) CFEs of mouse AT2 cells isolated from untreated or BLM-injured *Atg5*^*f/f*^ mice at day 10 after seeding (n = 4). (B) Representative micrographs of organoid cultures of mouse AT2 cells isolated from *Atg5*^*f/f*^, *Sftpc*^*CreER*^*;Atg5*^*f/f*^ (pretreated with tamoxifen), or *Nkx2*.*1*^*Cre*^*;Atg5*^*f/f*^ mice 14 days after BLM injury. Scale bar: 200 μm. (C and D) CFEs (C) and sizes (D) of organoid colonies from *Atg5*^*f/f*^, *Sftpc*^*CreER*^*;Atg5*^*f/f*^ (pretreated with tamoxifen), or *Nkx2*.*1*^*Cre*^*;Atg5*^*f/f*^ mice at day 14 after BLM injury (n = 4). (E and F) (E) Immunostaining of organoid colonies and (F) quantification of fractions of Ki67^+^pro-SPC^+^ cells in total pro-SPC^+^ cells in AT2 organoids (n = 4). Data are representative of three independent experiments with error bars representing the mean ± SD. ^∗^p < 0.05.

To assess the role of autophagy on AT2 cell proliferation *in vitro*, we performed 3D organoid culture of AT2 cells as we described previously (Chen et al., 2012). At day 14 after BLM, the CFE of surviving AT2 cells was clearly reduced relative to that of AT2 cells from PBS-treated mice (Figure 2A). At this time point, *Atg5*^−/−^ AT2 cells isolated from tamoxifen-treated *Sftpc*^*CreER*^*;Atg5*^*f/f*^ or *Nkx2*.*1*^*Cre*^*;Atg5*^*f/f*^ mice produced markedly fewer and smaller organoids than did AT2 cells isolated from *Atg5*^*f/f*^ lungs (Figures 2B–2D). Immunofluorescent staining of organoid cultures indicated that the ratio of Ki67^+^pro-SPC^+^ and pro-SPC^+^ cells was lower in cultures from tamoxifen-treated *Sftpc*^*CreER*^*;Atg5*^*f/f*^ or *Nkx2*.*1*^*Cre*^*;Atg5*^*f/f*^ mice relative to those from *Atg5*^*f/f*^ mice (Figures 2E and 2F). The expression of *Sftpc*, the gene encoding pro-SPC, was significantly reduced in cultures from *Nkx2*.*1*^*Cre*^*;Atg5*^*f/f*^ mice relative to *Atg5*^*f/f*^ mice, which was probably due to reduced organoid numbers (Figure S3A). Likewise, *Sftpb* and *Sftpd* were also reduced in AT2 cells in absence of Atg5 (Figure S3A). Under such conditions, the expression of the AT1 markers *T1α* and *Aqp5* remained unchanged in the absence of Atg5 (Figure S3B). These data suggested that autophagy sustains AT2 cell proliferation potential during BLM-induced injury.

### Autophagy Reprograms Metabolic Pathways in AT2 Cells in Response to BLM

**Figure 3:**
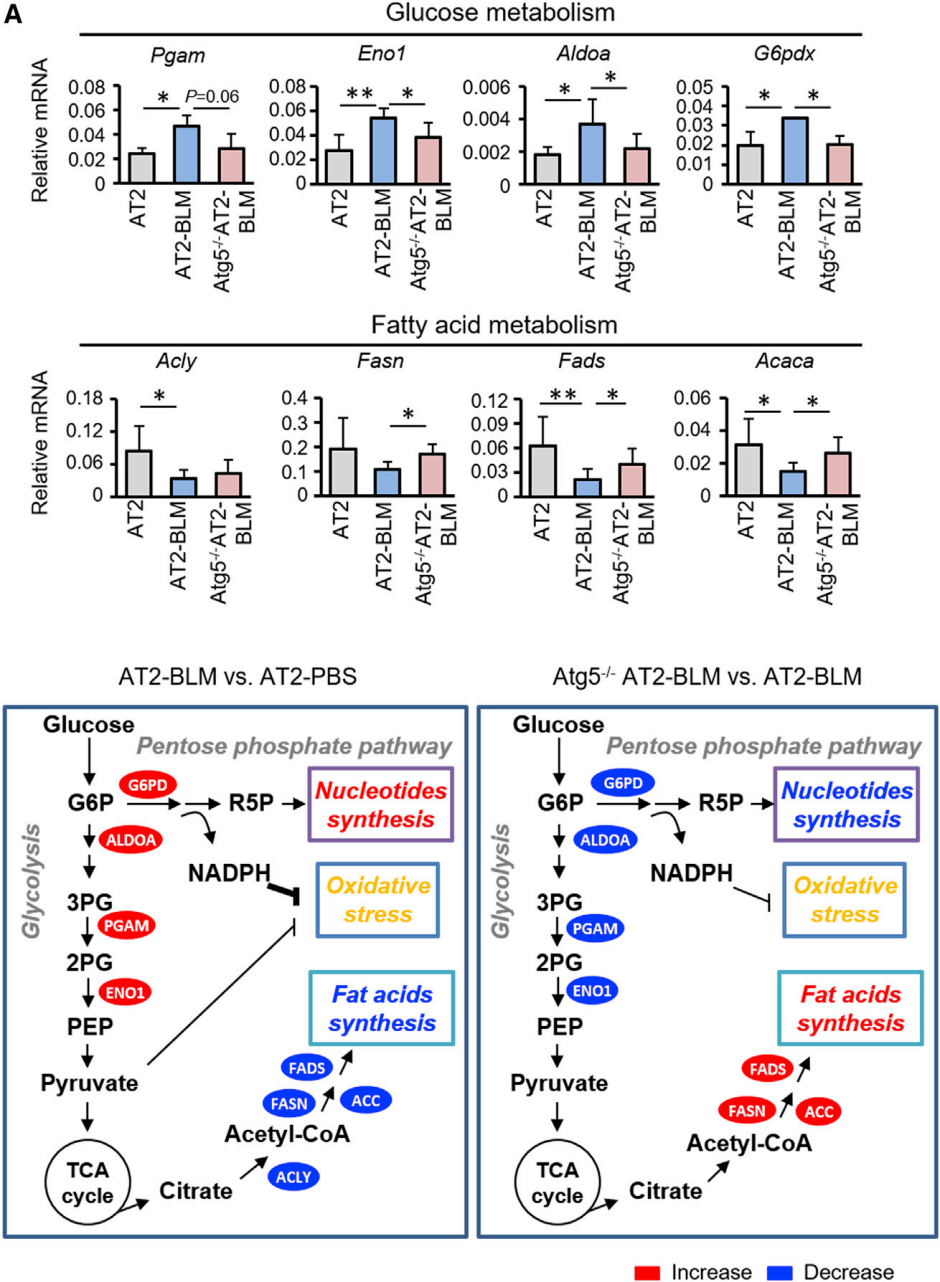
Autophagy Reprograms Metabolic Pathways in AT2 Cells in Response to BLM Challenge (A) qPCR validation of transcripts associated with glucose metabolism and fatty acid metabolism in AT2 cells (n = 3). Data are representative of three independent experiments with error bars representing the mean ± SD. ^∗^p < 0.05, ^∗∗^p < 0.01. (B) Schematic showing alterations in intermediates associated with glycolysis, pentose phosphate pathway, and synthesis of fatty acids in AT2 cells. Intermediates with increased and decreased expression are labeled in red and blue, respectively. G6P, glucose 6-phosphate; 3PG, 3-phosphoglyceric acid; 2PG, 2-phosphoglyceric acid; and PEP, phosphoenolpyruvate.

Autophagy is a cellular catabolic process that supports metabolism in response to stress. To identify metabolic pathways that are modulated by autophagy in AT2 cells during BLM-induced lung injury, RNA-seq and metabolic profiling were carried out with AT2 cells from *Atg5*^*f/f*^ mice treated with PBS (AT2), *Atg5*^*f/f*^ mice treated with BLM (AT2-BLM), and *Nkx2*.*1*^*Cre*^*;Atg5*^*f/f*^ mice treated with BLM (*Atg5*^−/−^ AT2-BLM) at day 14. Integrated transcriptome/metabolic profiling of significantly altered transcripts and metabolites was done, with reference to all metabolic pathways defined by the Kyoto Encyclopedia of Genes and Genomes (KEGG) (Figure S4; Tables S1–S4). Eight pathways that were altered in AT2 cells during BLM injury were regulated by autophagy (Figure S4). Among these, fatty acid synthesis, Wnt signaling, phosphatidylinositol signaling, and insulin signaling were suppressed by BLM injury (Figure S4). However, all four pathways were promoted in *Atg5*^−/−^ AT2 cells relative to wild-type (WT) AT2 cells during BLM injury (Figure S4). The pathways with increased expression, including glutathione metabolism, glycolysis, and pentose phosphate, were downregulated in *Atg5*^−/−^ AT2 cells (Figure S4). We used qPCR to validate the gene expression profiles of transcripts encoding metabolic enzymes associated with the glycolytic pathway. We found that transcripts, including phosphoglycerate mutase (*Pgam*), enolase 1 (*Eno1*), and aldolase A fructose-bisphosphate (*Aldoa*), had elevated expression in AT2-BLM relative to AT2-PBS (Figure 3A). Parallel to glycolysis, expression of glucose-6-phosphate dehydrogenase X-linked (*G6pdx*), the rate-controlling enzyme of the pentose phosphate pathway, was also increased by BLM injury (Figure 3A). Increased *G6pdx* expression could result in generation of increased nicotinamide adenine dinucleotide phosphate (NADPH). In contrast, expression of transcripts encoding enzymes involved in fatty acid metabolism, including ATP citrate lyase (*Acly*), fatty acid desaturase (*Fads*), and acetyl-coenzyme A carboxylase alpha (*Acaca*), was decreased in AT2-BLM relative to AT2-PBS controls (Figure 3A). Expression of *Fads*, *Acaca*, and *fatty acid synthase* (*Fasn*) recovered in the absence of *Atg5* (Figure 3A). These data suggested that the glycolytic and pentose phosphate pathways were promoted, but that the synthesis of fatty acids was repressed, in mouse AT2 cells during BLM injury (Figure 3B, left box). Thus, autophagy may serve as a switch between these two metabolic pathways during lung injury (Figure 3B, right box).

Similar transcriptome and metabolic profiling analyses were also conducted in WT versus *Atg5*^−/−^ AT2 cells (Tables S5 and S6), which showed that *Eno1* mRNA expression was increased and *Acly* and *Acaca* mRNA decreased in the absence of Atg5 at steady state (Figures S5A–S5D). However, there were no observable differences between WT AT2 cells and *Atg5*^−/−^ AT2 cells in terms of CFE, sizes of organoids, or *T1α* expression in organoids (Figure S5E). This finding was consistent with the observation that *Nkx2*.*1*^*Cre*^*;Atg5*^*f/f*^ mice exhibit normal alveolar development (Figure S1).

### Absence of Glucose Transporter 1 Blocks AT2 Cell Proliferation

**Figure 4:**
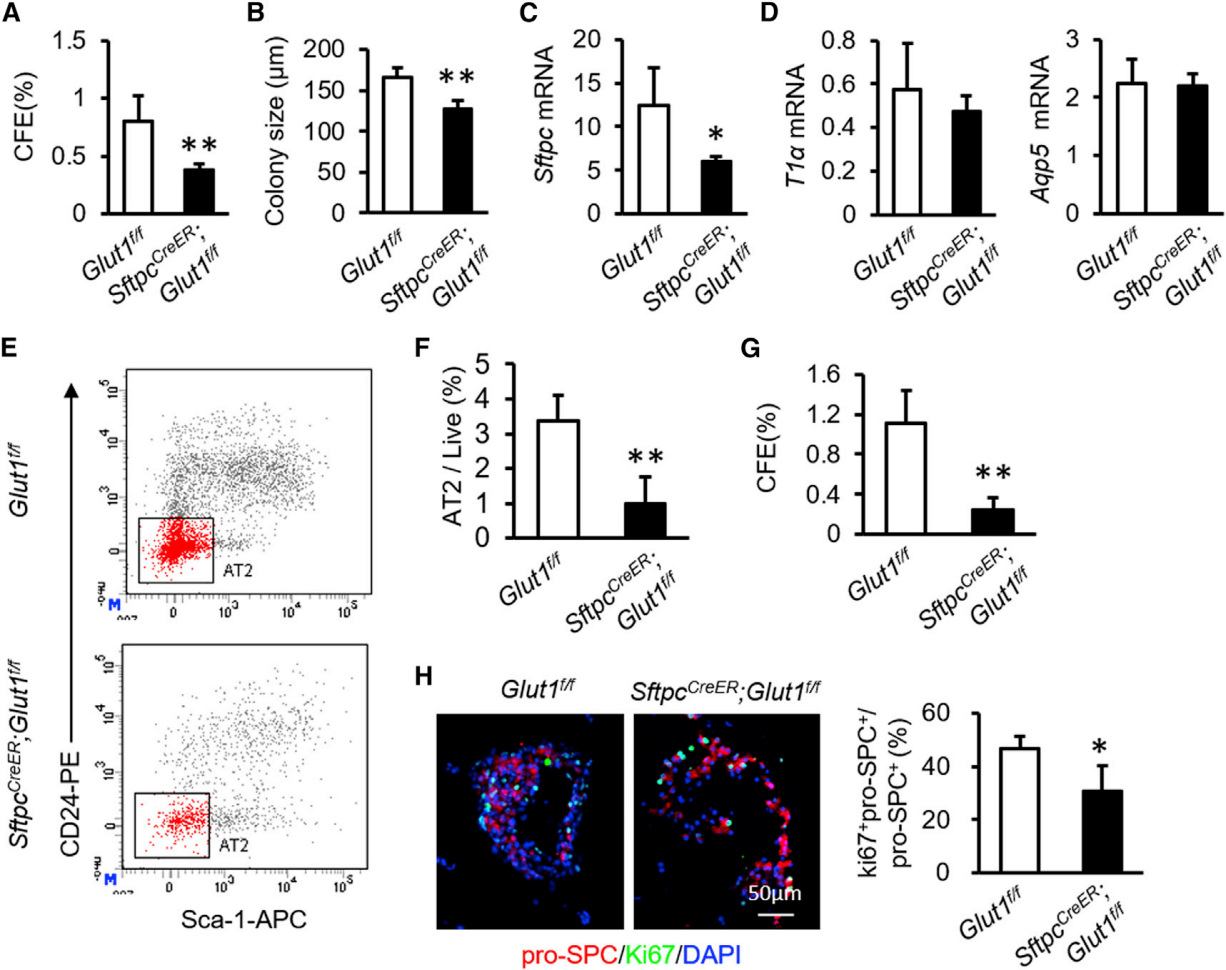
Genetic Deletion of Glut1 Inhibits AT2 Cell Proliferation (A) CFEs of AT2 cells isolated from *Atg5*^*f/f*^ or *Sftpc*^*CreER*^*;Glut1*^*f/f*^ mice at steady state (n = 5). (B) Colony sizes of organoid cultures of AT2 cells isolated from *Atg5*^*f/f*^ or *Sftpc*^*CreER*^*;Glut1*^*f/f*^ mice (n = 5). (C) *Sftpc* mRNA expression in organoid cultures (n = 5). (D) *T1α* and *Aqp5* mRNA expression in organoid cultures (n = 5). (E and F) Representative charts of flow cytometric analysis (E) and summarized abundance (F) of AT2 cells in *Sftpc*^*CreER*^*;Glut1*^*f/f*^ mice after BLM treatment relative to littermate controls (n = 5). (G) CFEs of AT2 cells isolated from *Atg5*^*f/f*^ or *Sftpc*^*CreER*^*;Glut1*^*f/f*^ mice after BLM treatment (n = 5). (H) Immunostaining of organoid cultures and quantification of Ki67^+^pro-SPC^+^ cells as a proportion of total pro-SPC^+^ cells in organoid cultures from *Atg5*^*f/f*^ or *Sftpc*^*CreER*^*;Glut1*^*f/f*^ mice after BLM treatment (n = 5). Data are representative of three independent experiments with error bars representing the mean ± SD. ^∗^p < 0.05, ^∗∗^p < 0.01.

We next investigated the role of glucose metabolism in the regulation of AT2 cell function. We selectively eliminated Glut1 in AT2 cells by establishing *Sftpc*^*CreER*^*;Glut1*^*f/f*^ mice. Tamoxifen treatment resulted in about 50% reduction in *Glut1* expression in AT2 cells from *Sftpc*^*CreER*^*;Glut1*^*f/f*^ mice (Figure S6). AT2 cells isolated from tamoxifen-treated *Sftpc*^*CreER*^*;Glut1*^*f/f*^ mice generated fewer and smaller colonies (Figures 4A and 4B). Consistent with these data, the expression of *Sftpc* was significantly reduced in *Glut1*^*−/−*^ AT2 cultures relative to littermate controls (Figure 4C). *T1α* and *Aqp5* expression remained unchanged in cultures of *Glut1*^*−/−*^ versus WT AT2 cells (Figure 4D). Flow cytometry of total disassociated lung cells indicated that fractions of AT2 cells in the total live cell population were lower in *Sftpc*^*CreER*^*;Glut1*^*f/f*^ mice treated with tamoxifen relative to *Glut1*^*f/f*^ mice after BLM exposure (Figures 4E and 4F). CFEs of the surviving AT2 cells isolated from tamoxifen-treated *Sftpc*^*CreER*^*;Glut1*^*f/f*^ mice were significantly decreased from those produced by littermate controls (Figure 4G). Consistent with these data, the ratios of Ki67^+^pro-SPC^+^ and pro-SPC^+^ cells were lower in organoid cultures from *Sftpc*^*CreER*^*;Glut1*^*f/f*^ mice relative to those from *Glut1*^*f/f*^ mice after BLM treatment (Figure 4H). These data suggest that proliferation, but not differentiation, of AT2 cells requires Glut1 function.

### Glycolysis and Pentose Phosphate Pathways Are Essential for AT2 Cell Proliferation

**Figure 5:**
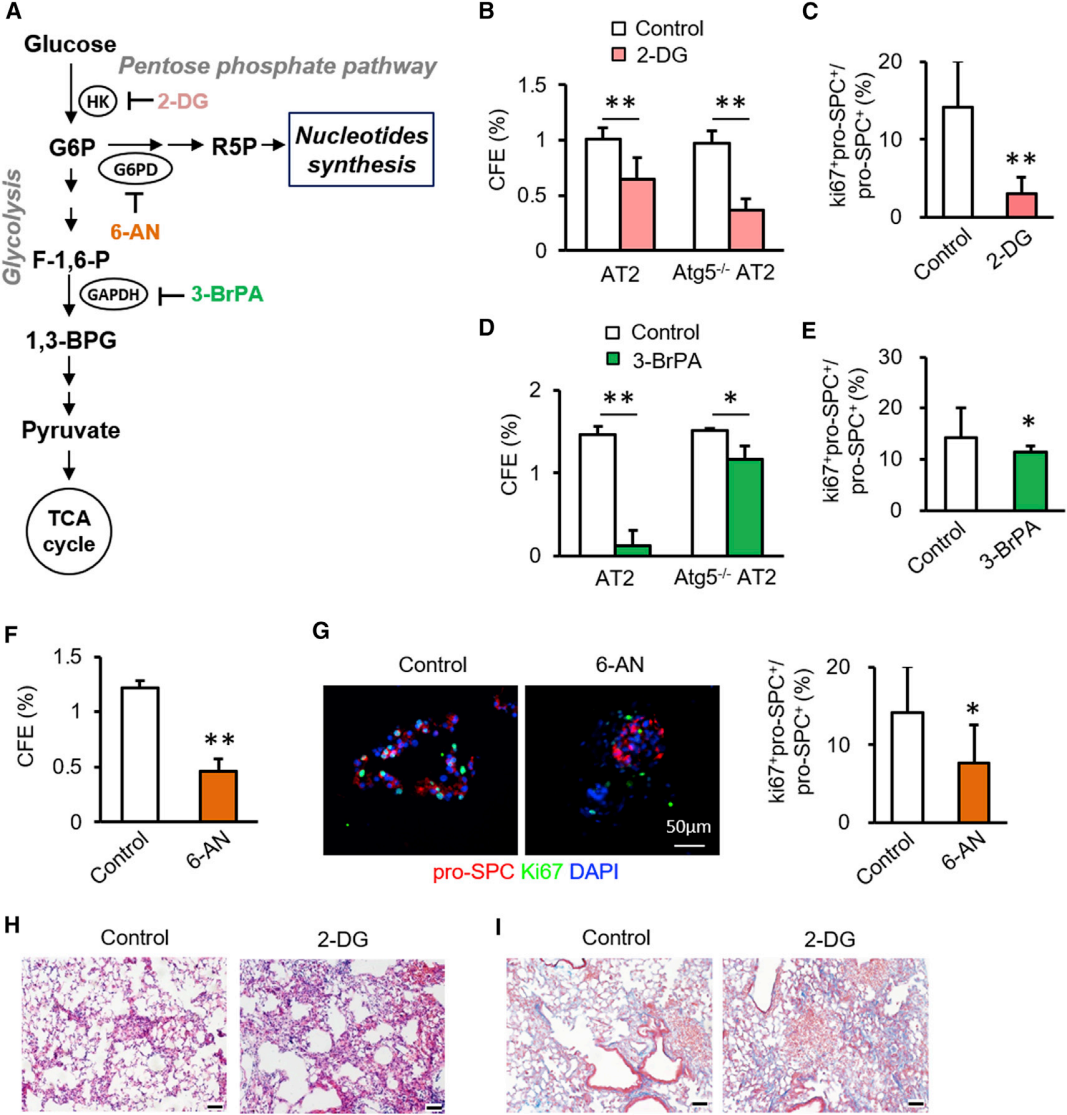
AT2 Cell Proliferation Requires Glucose Metabolism (A) Schematic of intermediates and their inhibitors associated with glycolytic and pentose phosphate pathways. (B) CFE of mouse AT2 and *Atg5*^−/−^ AT2 cells with or without 2-DG (n = 5). (C) Proportions of Ki67^+^pro-SPC^+^ cells in total pro-SPC^+^ cells in the presence and absence of 2-DG (n = 5). (D) CFE of AT2 and *Atg5*^−/−^ AT2 cultures with and without 3-BrPA (n = 5). (E) Proportions of Ki67^+^pro-SPC^+^ cells in total pro-SPC^+^ cells in organoid cultures in the presence and absence of 3-BrPA (n = 5). (F) CFE of mouse AT2 cells cultures with or without 6-AN (n = 5). (G) Immunostaining of organoid cultures and quantification of Ki67^+^pro-SPC^+^ cells in total pro-SPC^+^ cells in the presence and absence of 6-AN (n = 5). (H and I) After intratracheal instillation of BLM, *Atg5*^*f/f*^ mice were intraperitoneally injected with PBS or 100 mg/kg 2-DG. At 14 days after BLM, lungs were harvested for hematoxylin/eosin (H) and Masson trichrome (I) staining. Scale bar: 50 μm. Data are representative of two independent experiments with error bars representing the mean ± SD. ^∗^p < 0.05, ^∗∗^p < 0.01.

After its entry into cells, glucose is phosphorylated by hexokinase (HK) to generate G6P, which has two metabolic fates: the glycolytic pathway or the pentose phosphate pathway (Figure 5A). Therefore, we determined whether AT2 cell proliferation is influenced by the metabolic fate of glucose using selective inhibitors of enzymes involved in these two metabolic pathways. 2-Deoxy-D-glucose (2-DG), an inhibitor of HK, increased CFEs slightly at 0.5 mM, but decreased CFEs at 1.0 mM (Figure 5B). Treatment with 2-DG led to a similar reduction in CFEs of *Atg5*^−/−^ AT2 cells isolated from *Nkx2*.*1*^*Cre*^*;Atg5*^*f/f*^ mice (Figure 5B). The ratio of Ki67^+^pro-SPC^+^ to pro-SPC^+^ cells was lower in the presence of 2-DG at 1.0 mM (Figure 5C). The glycolytic inhibitor 3-bromopyruvate (3-BrPA) decreased CFEs in a dose-dependent fashion (Figure 5D). Treatment with 3-BrPA led to a reduction to a lesser extent in CFEs of *Atg5*^−/−^ AT2 cells isolated from *Nkx2*.*1*^*Cre*^*;Atg5*^*f/f*^ mice as compared with AT2 cells, suggesting that the effect of 3-BrPA on the CFEs of AT2 cells is mediated through both autophagy-dependent and autophagy-independent pathways (Figure 5D). The ratio of Ki67^+^pro-SPC^+^ to pro-SPC^+^ cells was lower in organoid cultures in the presence of 100 μM 3-BrPA (Figure 5E). CFEs were also decreased in the presence of 6-aminonicotinamide (6-AN), an inhibitor of G6PD, at 50 μM (Figure 5F). Relative to control cultures, the fraction of Ki67^+^pro-SPC^+^ over pro-SPC^+^ cells was lower in the presence of 50 μM 6-AN (Figure 5G). Relative to control mice, intraperitoneal administration of 2-DG produced a significantly enhanced fibrotic response, as illustrated by distorted alveolar structure and enhanced trichrome staining 14 days after BLM challenge (Figures 5H and 5I). These data suggest that glucose metabolism is beneficial for AT2 cell proliferation.

### Inhibition of the Fatty Acid Synthesis Pathway Promotes AT2 Cell Proliferation

**Figure 6:**
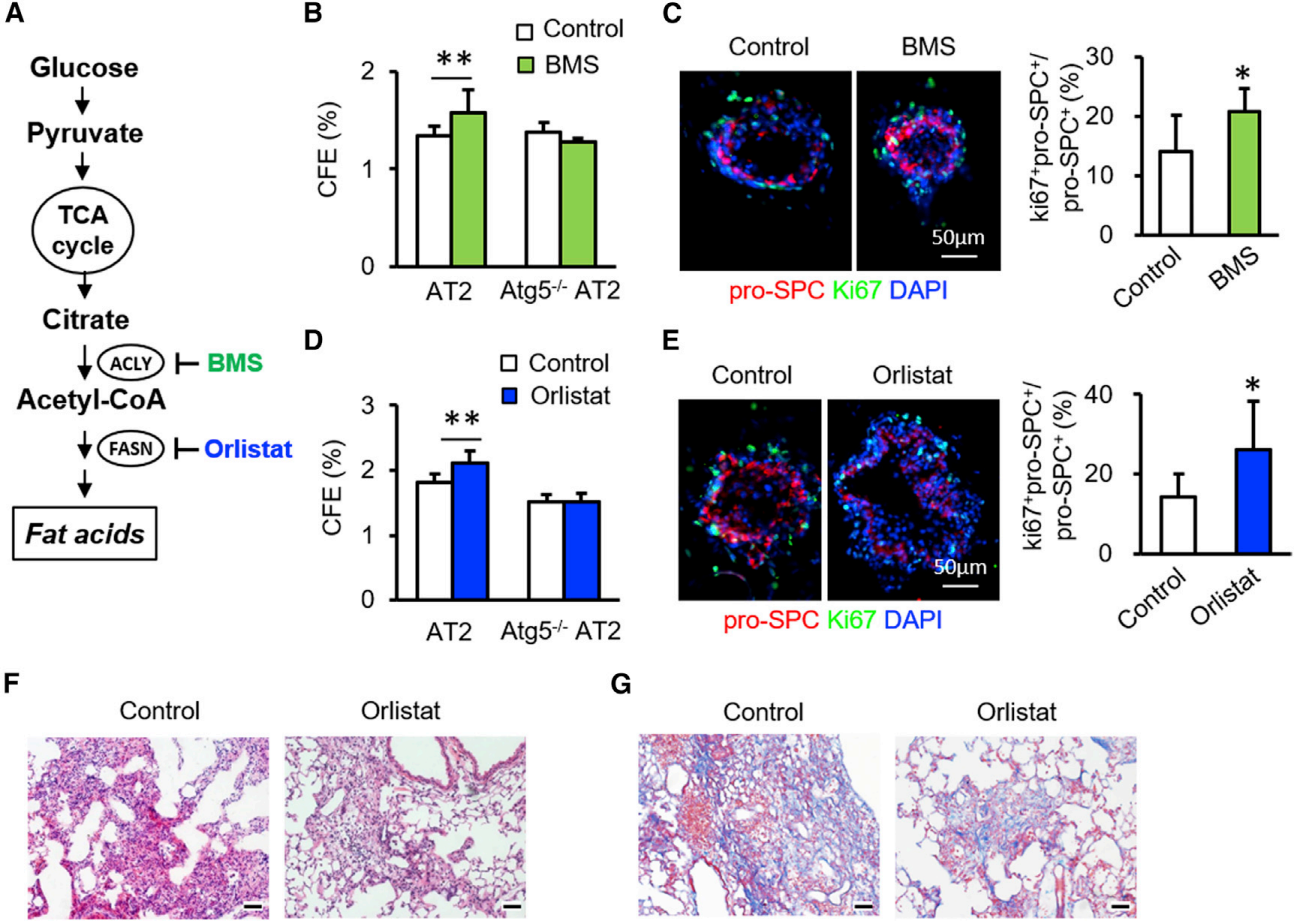
Fatty Acid Synthesis Pathway Negatively Influences AT2 Cell Proliferation (A) Schematic of intermediates and their inhibitors in fatty acid synthesis pathways. (B) CFE of mouse AT2 cultures with and without BMS (n = 5). (C) Immunostaining of organoid cultures and quantification of Ki67^+^pro-SPC^+^ cells in total pro-SPC^+^ cells in organoid cultures in the presence and absence of BMS (n = 5). (D) CFE of AT2 cell cultures with and without orlistat (n = 5). (E) Immunostaining of organoid cultures and proportions of Ki67^+^pro-SPC^+^ cells in total pro-SPC^+^ cells in organoid cultures in the presence and absence of orlistat (n = 5). (F and G) After intratracheal instillation of BLM, *Nkx2*.*1*^*Cre*^*;Atg5*^*f/f*^ mice were intraperitoneally injected with PBS or orlistat. At 14 days after BLM, lungs were harvested for hematoxylin/eosin (F) and Masson trichrome (G) staining. Scale bar: 50 μm. Data are representative of two independent experiments with error bars representing the mean ± SD. ^∗^p < 0.05, ^∗∗^p < 0.01.

Citrate is converted to acetyl-CoA by Acly, which is then utilized by fatty acid synthase to produce fatty acids (Figure 6A). We next investigated the regulation of mouse AT2 cell proliferation by the fatty acid synthesis pathway. The Acly inhibitor BMS-303141 increased CFEs of AT2 cells at 50 μM (Figure 6B). This BMS-induced increase in CFE was abolished in *Atg5*^−/−^ AT2 cells isolated from *Nkx2*.*1*^*Cre*^*;Atg5*^*f/f*^ mice (Figure 6B). The ratios of Ki67^+^pro-SPC^+^ to pro-SPC^+^ cells were significantly increased in AT2 cell organoid cultures with BMS relative to control cultures (Figure 6C). Similar to BMS, the FASN inhibitor orlistat enhanced CFE (Figure 6D). The orlistat-induced increase in CFEs also was abolished in *Atg5*^−/−^ AT2 cells isolated from *Nkx2*.*1*^*Cre*^*;Atg5*^*f/f*^ mice (Figure 6B). Consistent with this, ratios of Ki67^+^pro-SPC^+^ to pro-SPC^+^ cells were increased by orlistat (Figure 6E). Because fatty acid metabolism was promoted in AT2 cells in the absence of Atg5 (Figure 3B). We observed that orlistat appeared to counteract the effects of autophagy loss on BLM-induced lung injury (Figures 6F and 6G). These data suggest that the fatty acid synthesis pathway has a negative influence on mouse AT2 cell proliferation.

### Autophagy Prevents Oxidative Stress-Induced Impairment of Proliferation of AT2 Cells

**Figure 7:**
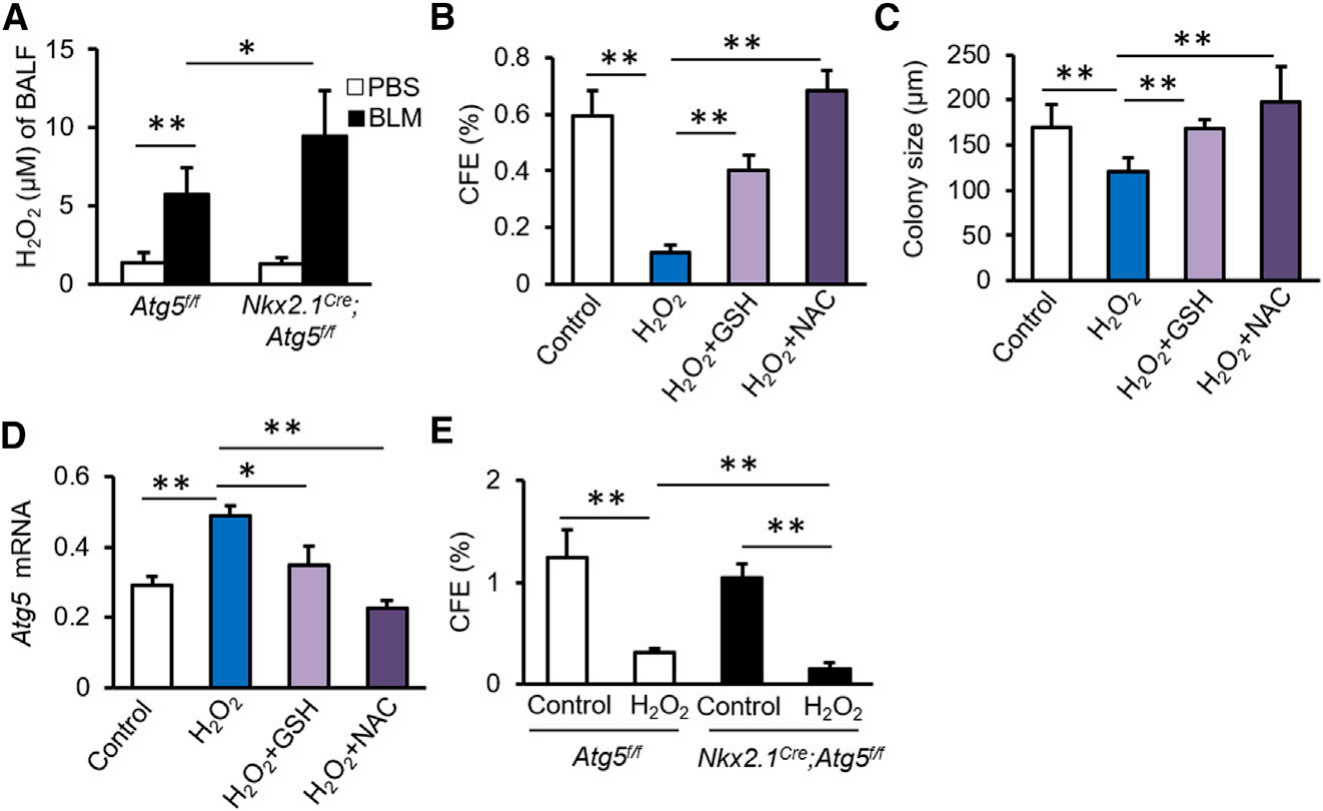
Autophagy Sustains AT2 Cell Proliferation by Repressing H_2_O_2_ Production during Injury (A) H_2_O_2_ levels in BALF from *Atg5*^*f/f*^ and *Nkx2*.*1*^*cre*^*;Atg5*^*f/f*^ mice 14 days after BLM injury (n = 5). (B) CFE of AT2 cells cultured in the presence of H_2_O_2_, H_2_O_2_ + GSH, or H_2_O_2_ + NAC (n = 6). (C) Diameters of colonies under conditions described in (B) (n = 6). (D) *Atg5* gene expression in organoid cultures under indicated conditions (n = 6). (E) CFE of AT2 cells isolated from control *Atg5*^*f/f*^ and *Nkx2*.*1*^*Cre*^*;Atg5*^*f/f*^ mice in the presence or absence of H_2_O_2_ (n = 5). Data are representative of two independent experiments with error bars representing the mean ± SD. ^∗^p < 0.05, ^∗∗^p < 0.01.

The pentose phosphate pathway is an important source of antioxidants, as a result of its generation of NADPH. The transcriptome and metabolome analyses indicated that *Atg5* loss reduced pentose phosphate pathway activity, suggesting that autophagy may repress oxidative stress during lung injury. To further examine this, we measured the concentrations of H_2_O_2_ in bronchoalveolar lavage fluid (BALF). H_2_O_2_ levels were elevated in mice treated with BLM relative to those of control mice (Figure 7A). As predicted, *Atg5* loss in lung epithelium further promoted the production of H_2_O_2_ in BALF (Figure 7A). H_2_O_2_ significantly decreased CFE and reduced the size of AT2 organoid colonies (Figures 7B and 7C). The addition of the antioxidants glutathione (GSH) or N-acetylcysteine (NAC) counteracted the effect of H_2_O_2_ (Figures 7B and 7C). H_2_O_2_ had little effect on AT2 cell differentiation, as demonstrated by the expression of *T1α* and *Aqp5* mRNAs in organoid cultures (Figure S7). *Atg5* expression was elevated in organoid cultures in the presence of H_2_O_2_ (Figure 7D), but this elevation was abolished by addition of GSH or NAC (Figure 7D). In the presence of H_2_O_2_, *Atg5*^−/−^ AT2 cells displayed decreased CFE relative to control AT2 cells (Figure 7E). These data suggest that autophagy sustains AT2 cell proliferation by eliminating oxidative stress during BLM-induced lung injury.

## Discussion

Severe injury to the alveolar epithelium can lead to respiratory failure. Despite a growing appreciation for the protective role of autophagy in lung injury, whether it modulates the regenerative machinery in alveoli remains unknown, as do the underlying mechanisms by which it might do so. Our data demonstrate that epithelial autophagy is dispensable for lung development. After injury, however, autophagy is promoted in surviving AT2 cells to sustain proliferation and ameliorate fibrotic progression. Combined transcriptome and metabolome analyses in AT2 cells indicated that autophagy protects from BLM-induced lung injury by changing glucose and lipid metabolism.

Increased reactive oxygen species (ROS) have been described in human lung fibrosis patients and in BLM-induced mouse models of lung injury (Cheresh et al., 2013, Mouratis and Aidinis, 2011). A high level of ROS would likely induce AT2 cell senescence in lung fibrotic injury (Lehmann et al., 2017). We observed that high levels of H_2_O_2_ significantly abrogated the proliferative capacity of AT2 cells but had no effect on AT1 differentiation. Our data suggest that ROS may induce autophagy activation, which in turn limits ROS production and prevents ROS-induced lung injury. *In vitro* organoid cultures demonstrated that addition of the antioxidants N-acetylcysteine and superoxide dismutase counteracted the deleterious effects of ROS on AT2 cell proliferation. We propose that antioxidants are beneficial for epithelial repair and regeneration in the lung. Autophagy may be induced by ROS as feedback to limit ROS-induced epithelial injury.

Autophagy has been shown to be induced after birth and *Atg5* knockout mice die as newborns (Cheong et al., 2014, Kuma et al., 2004). In this study, *Nkx2*.*1*^*Cre*^*;Atg5*^*f/f*^ mice were viable, with normal lung structure and surfactant expression, suggesting that epithelial autophagy is dispensable for lung development. Transcriptome analysis indicated that expression of the glycolysis enzyme ENO1 is elevated in AT2 cells in the absence of Atg5, suggesting that autophagy may limit glucose use. This may explain the accumulation of glycogen in *Atg5*-deficient AT2 cells at steady state (Cheong et al., 2014).

During BLM-induced lung injury, glucose metabolism was promoted in AT2 cells. The conditional deletion of *Glut1* reduced proliferation of AT2 cells in organoid cultures and impaired recovery of AT2 cells *in vivo* during BLM-induced injury. We further found that the glycolytic and pentose phosphate pathways were enhanced in AT2 cells under such an injury condition.

Enhanced glycolysis may increase glycolytic ATP production, which is important for rapidly proliferating cells (Moussaieff et al., 2015). Glycolysis also shunts into the pentose phosphate pathway, limiting ROS production and providing nucleotides for cell proliferation (Vander Heiden et al., 2009). During injury repair after BLM injury, surviving AT2 cells take advantage of these two pathways to regenerate alveolar epithelium. The effects of glucose metabolism on AT2 cells appear to be mediated by both autophagy-dependent and -independent mechanisms. Glucose has recently been shown to stimulate intestinal epithelial crypt proliferation by inducing glycolysis (Zhou et al., 2018). Blockade of pyruvate entry into mitochondria increased proliferation and expanded intestinal stem cell compartments (Schell et al., 2017). Thus glycolysis, or its intermediates promote stem cell proliferation. Future studies are needed to investigate if alveolar epithelial regeneration can also be modulated by altering pyruvate metabolism.

In contrast to glucose metabolism, acetyl-CoA utilization in fatty acid synthesis negatively affects AT2 cell proliferation. Notably, the fatty acid synthesis pathway was repressed in AT2 cells during BLM-induced lung injury. These data suggest that the surviving AT2 cells regenerate alveolar epithelium after injury by repressing lipid metabolism in addition to promoting glucose metabolism. Loss of Atg5 resulted in reduced glucose metabolism, but increased lipid metabolism, suggesting that autophagy may regulate switching between these two metabolic pathways. During injury repair, autophagy is induced in the surviving AT2 cells to switch from lipid metabolism to glucose metabolism to accelerate alveolar epithelial regeneration. Autophagy is an essential cellular process that recycles cytoplasmic components (Herzig and Shaw, 2018). Lipid metabolic pathways have been reported to inversely regulate autophagy (Marino et al., 2014). Thus, lipid metabolism and autophagy could coordinate in the fashion of positive feedback in alveolar regeneration during stress conditions. Fatty acid is generally utilized to synthesize at least three kinds of lipids, including sphingolipids and eicosanoids, in addition to phospholipids that are essential for growth-associated lipid synthesis for cell membranes (Baenke et al., 2013). Future studies are needed to test whether or not synthetic pathways of sphingolipids and eicosanoids play a negative role in regulating alveolar epithelial regeneration.

In summary, the present findings reveal a crucial role for autophagy in alveolar epithelial regeneration. Basal autophagy is dispensable for lung development and AT2 cell functions. After injury repair, autophagy is induced in the surviving AT2 cells for their proliferation. AT2 cell proliferation is positively regulated by glucose metabolism but is negatively affected by lipid metabolism. Autophagy switches lipid metabolism to glucose metabolism to secure epithelial regeneration after injury. Our results elucidate an important link between autophagy, metabolism, and alveolar progenitor cell function, and could provide a clue to better understand lung homeostasis and injury repair.

## Experimental Procedures

### Mice

C57BL/6J mice were purchased from Beijing Huafukang Bioscience. *Nkx2*.*1-Cre* mice were purchased from The Jackson Laboratory (Bar Harbor, ME). *Atg5*^*f/f*^ mice were originally obtained from RIKEN BRC through the National Bio-Resource Project of the MEXT in Japan. *Nkx2*.*1-Cre* mice were crossed with *Atg5*^*f/f*^ mice to generate *Nkx2*.*1*^*Cre*^*;Atg5*^*f/f*^ mice. *Sftpc*^*CreER*^ mice were purchased from The Jackson Laboratory (stock no. 028054). *Atg5*^*f/f*^ mice were crossed with *Sftpc*^*CreER*^ mice to generate *Sftpc*^*CreER*^*;Atg5*^*f/f*^ mice. *Glut1*^*f/f*^ mice, generated as described previously (Young et al., 2011), were crossed with *Sftpc*^*CreER*^ mice to generate *Sftpc*^*CreER*^*;Glut1*^*f/f*^ mice. Tamoxifen was injected intraperitoneally at 50 mg/kg once daily for 5 days to induce knockdown of Atg5 or Glut1 in mouse AT2 cells. All mice were housed in a specific pathogen-free facility at Tianjin University Haihe Hospital. All mice were exposed to an alternating 12-h light/dark cycle and had free access to food and water. Age- and sex-matched mice were randomly grouped. Eight- to 12-week-old male mice were used in BLM-induced lung injury experiments. All mice were treated with strict adherence to the protocol (no. 2015HHLL06) approved by the Tianjin University Haihe Hospital Animal Care and Use Committee.

### BLM-Induced Lung Injury

BLM treatment was performed as described previously (Chen et al., 2012). In brief, mice were anesthetized and then intratracheally instilled with 2 U/kg BLM (Nippon Kayaku, Tokyo, Japan) with a 25-gauge needle inserted between the cartilaginous rings of the trachea. Control animals received PBSalone. Mice were allowed to recover after the tracheostomy site was sutured. At designated times after BLM instillation, mice were euthanized, and lung tissues were harvested for immunohistochemistry or RNA isolation. To harvest BALF, the trachea was cannulated and lavaged two times with 1-mL sterile PBS at room temperature. Cells were pelleted at 600 × *g* for 15 min and supernatants were collected for hydrogen peroxide (H_2_O_2_) measurement.

### Fibroblast Cultures

The MLg2908 mouse lung fibroblast cell line (CCL-206, ATCC) was cultured in DMEM (Gibco, Franklin Lakes, NJ) supplemented with 10% fetal bovine serum (FBS) (Gibco), 100 IU/mL penicillin, and 100 μg/mL streptomycin at 37°C in a humidified atmosphere containing 5% CO_2_.

### Mouse Lung Disassociation and Flow Cytometry

Lung single-cell suspensions were generated by elastase digestion and stained for FACS as described previously (Chen et al., 2012). In brief, mouse lungs were perfused once with 1 mL PBS and minced in a solution with elastase (4 U/mL; Worthington Biochemical Corporation, Lakewood, NJ), followed by incubation with DNase I (100 U/mL; Sigma-Aldrich, St. Louis, MO) for 15 min at 37°C. The resulting cell suspension was filtered through a 100-μm cell strainer (Falcon; BD Biosciences, San Jose, CA) for flow cytometry. Red blood cell lysis buffer was added to remove erythrocytes. Cell pellets were resuspended in Hank's balanced salt solution (HBSS) supplemented with 2% FBS, 10 mM HEPES, 0.1 mM EDTA, 100 IU/mL penicillin, and 100 μg/mL streptomycin (HBSS-plus). Flow cytometry was performed with primary antibodies against CD31-biotin, CD34-biotin, CD45-biotin, CD24-phycoerythrin (PE), anti-epithelial cellular adhesion molecule (EpCAM)-PE-Cy7, and Sca-1-allophycocyanin. The secondary antibody was against streptavidin. All antibodies were from eBioscience (San Diego, CA). Cells were stained for approximately 45 min in the dark on ice. After one wash with HBSS-plus, 7-amino-actinomycin D was added to label dead cells. Flow cytometry analysis was performed using single-color controls for compensation and established gating strategies based on isotype-negative controls on a FACSAria III sorter (BD Immunocytometry Systems, San Jose, CA). Mouse AT2 cells, defined as CD31^−^CD34^−^CD45^−^(Lin^−^) EpCAM^+^CD24^−^Sca-1^−^, were sorted in HBSS-plus for functional experiments, including 3D organoid cultures. Alternatively, cells were harvested and lysed with TRIzol reagent for RNA analysis, or with the miRNA Isolation Kit (1561; Ambion, Austin, TX) for gene sequencing, or frozen in liquid nitrogen for metabolite analysis by gas chromatography-mass spectrometry (GC-MS).

### Organoid Cultures

Flow-sorted mouse Lin^−^EpCAM^+^CD24^−^Sca-1^−^ AT2 cells were co-cultured with MLg fibroblasts as described previously (Chen et al., 2012). In brief, 2 × 10^4^ AT2 cells and 2 × 10^5^ MLg fibroblasts were mixed in 100 μL Matrigel/basic medium (1:1). The basic medium consisted of DMEM/F12 medium (Cellgro, Manassas, VA), 10% FBS, 1% insulin-transferrin-selenium supplement (Sigma-Aldrich), 100 IU/mL penicillin, and 100 μg/mL streptomycin. The cell suspension was seeded in 24-well Transwell filter inserts (Becton Dickinson, Franklin Lakes, NJ) in a 24-well flat-bottom culture plate containing 410 μL basic culture medium (BM + SB431542). Cultures were incubated at 37°C in a humidified atmosphere of 5% CO_2_, with daily replacement of the medium. Colonies were visualized with an IX73 inverted fluorescence microscope (Olympus, Tokyo, Japan). Colonies with diameters ≥100 μm were counted and the CFE was calculated as the number of colonies in each insert as a percentage of the number of input AT2 cells at day 10 after seeding. For optimal embedding and sectioning, the Matrigel disks were fixed with freshly made 4% paraformaldehyde in PBS for 1 h at 4°C and embedded in O.C.T. medium after a brief wash with PBS. Embedded colony disks were sliced at a thickness of 5 μm for staining. For gene expression analysis, TRIzol (Invitrogen, Carlsbad, CA) was added to the Matrigel disks and RNA was extracted following the manufacturer's instructions.

### RNA Extraction and qPCR

Total RNA was extracted from AT2 cells or lung tissues with TRIzol reagent (Invitrogen) following the manufacturer's instructions. For qPCR, 0.2 μg total RNA was reverse transcribed with SuperScript III reagents (Invitrogen) with oligo-d(T) (Takara Bio, Shiga, Japan) and random hexamer (Takara Bio) primers. Real-time qPCR was performed using SYBR Green Supermix (Applied Biosystems, Foster City, CA) with the Light Cycler 96 Real-Time PCR system (Roche Diagnostics, Indianapolis, IN). PCR conditions were 95°C for 2 min, 40 cycles of 95°C for 10 s, 60°C for 20 s, and 72°C for 20 s. The relative expression levels of each gene of interest were normalized using the level of E-cadherin in the same sample. Fold changes in target-gene expression were calculated using the 2^ΔΔCt^ method. Primer sequences are shown in Table S7.

### RNA-Seq

RNA was extracted from AT2 cells sorted from PBS-treated WT mice (AT2-PBS), BLM-exposed WT mice (AT2-BLM), and BLM-exposed *Nkx2*.*1*^*Cre*^*;Atg5*^*f/f*^ mice (*Atg5*^−/−^AT2 -BLM) using the mirVana miRNA Isolation Kit (Ambion). RNA integrity was assessed using a model 2100 Bioanalyzer (Agilent Technologies, Santa Clara, CA), and samples with RNA integrity >7 were chosen for library preparation. Libraries were constructed using a TruSeq Stranded mRNA Kit (Illumina, San Diego, CA), followed by the Agencourt AMPure XP with an Illumina HiSeq 2500. FASTQ files were assessed for quality using the FastQC program (Babraham Bioinformatics, Cambridge, UK), and low-quality reads were removed with Trimmomatic software. FASTQ files were aligned against the mouse reference genome (mm9) using the STAR aligner. Gene counts are represented as counts per fragment per kilobase of exon per million fragments mapped using Cufflinks software and normalized with DESeq software. Significantly differentially expressed genes were selected using p value < 0.05 and fold change ≥ 1.4. Principal-component analysis and heatmaps were generated in R. We conducted analyses of enriched KEGG pathways using the MetaboAnalyst 4.0 webservice (http://www.metaboanalyst.ca). The accession number for RNA-seq data reproted in this paper is GEO: GSE143212.

### Metabolomic Analysis

AT2 cells were added to 1.5-mL centrifuge tubes with 1 mL methanol and 20 μL internal standard containing 0.3 mg/ml L-2-chloro-phenylalanine. After addition of chloroform (200 μL), samples were processed for ultrasonic extraction for 20 min. Extracts were cleared at 13,000 × *g* for 10 min at 4°C. The resultant supernatants were transferred into glass vials and the contents evaporated. We added 80 μL methoxyamine hydrochloride dissolved in pure pyridine (15 mg/mL) to each vial, vortexed for 2 min, and placed the vials into a 37°C shaker-incubator to allow oxidation for 90 min. To each sample, 80 μL of N,O-bis(trimethylsilyl) trifluoroacetamide containing 1% trimethylsilyl chloride-derived reagents and 20 μL n-hexane was added. Each sample was vortexed for 2 min and reactions were allowed to proceed for 60 min at 70°C. Samples were cooled at room temperature for 30 min and 1-μL aliquots used for GC-MS analysis (Agilent Technologies). Separation was carried out on a nonpolar DB-5 capillary column (30 m × 250 μm; Agilent Technologies) with high-purity helium as the carrier gas at a constant flow rate of 1.0 mL/min. The GC temperature programming began at 60°C, continued with 8°C/min oven temperature ramped to 125°C, then 5°C/min to 210°C, 10°C/min to 270°C, and 20°C/min to 305°C. The electron impact ion source was held at 230°C with a filament bias of 70 eV.

GC-MS data from GC-MS was analyzed with Chroma TOF software (v. 4.34, LECO Corporation, Saint Joseph, MI). In brief, the dataset was normalized using the sum intensity of the peaks in each sample, then imported into the SIMCA software package (v.14.0; Umetrics, Umeå, Sweden). Principal-component analysis and (orthogonal) partial least-squares-discriminant analysis (OPLS-DA) were carried out to visualize the metabolic differences between groups. We used variable importance in the projection (VIP) to rank the overall contribution of each variable to the OPLS-DA model and considered variables with VIP > 1.0 as differential variables. We determined metabolites with both multivariate and univariate statistical significance (VIP > 1.0 and p < 0.05) as differential metabolites. The variation factor of different metabolites in the two groups was calculated as fold change. Joint pathway analysis of differential metabolites and transcripts identified in RNA-seq was performed using webservice MetaboAnalyst 4.0 (http://www.metaboanalyst.ca).

### Immunofluorescence

O.C.T.-embedded, 5-μm-thick cryosections of colonies were processed for antigen retrieval in citric acid (10 mM [pH 6]), followed by blocking with 5% BSA in 0.2% Triton X-/PBS for 30 min at room temperature. Primary antibodies were incubated overnight at 4°C: pro-SPC (1:200, Millipore, Billerica, MA), anti-Ki67 (1:200, eBioscience). Fluorochrome-conjugated secondary antibody (1:200, Invitrogen) was incubated at room temperature for 120 min. After washing with PBS, sections were mounted with Fluoromount G containing DAPI. Stained sections were imaged using a TCS SP5 confocal microscope (Leica, Wetzlar, Germany) or an IX73 inverted fluorescent microscope (Olympus, Tokyo, Japan). Three or more fields were picked randomly from each colony section for analysis.

### Hematoxylin and Eosin Staining

Lung sections were deparaffinized twice for 10 min with xylene and rehydrated through a series (100%, 95%, and 75%) of alcohol solutions followed by distilled water (10 min each). Rehydrated sections were stained in hematoxylin (322350; ZSQB-BIO, Beijing, China) for 3 min, rinsed in warm water for 10 min, dehydrated twice through 95% alcohol (2 min each), stained in eosin solution (ZLI9613; ZSQB-BIO) for 30 s, dehydrated through 95% and 100% alcohol solutions (3 min each), and cleared in xylene (5 min twice). Slides were mounted with neutral balsam and imaged using an IX73 microscope. Three or more fields were picked randomly from each colony section for analysis.

### Masson Trichrome Staining

Rehydrated sections received a drop of Masson compound stain solution for 5 min. The dye was removed with distilled water. A drop of phosphomolybdic acid stain was added for 5 min and then removed. A drop of aniline blue was added, left for 5 min, and rinsed with distilled water. A drop of differentiation liquid was added for 30–60 s. Sections were dehydrated using 95% and 100% ethanol (3 min each) and cleared using two washes with xylene (5 min each). Slides were mounted with neutral balsam and imaged using an IX73 microscope. Three or more fields were picked randomly from each colony section for analysis.

### Statistical Analyses

Data are representative of three or more independent experiments and are expressed as means ± SD. Student's t test was adopted to calculate p values between experimental and control groups. A p value < 0.05 was considered statistically significant. Mice were randomly assigned to control or experimental groups, with inclusion criteria dependent upon sex and age. Sample size for mouse experiments was determined based on pilot experiments. Outside of technical errors, no mice were excluded from the statistical analyses. Data were normally distributed, with no significant variance between groups.

## Author Contributions

X.L., Q.W., X.S., and H.C. designed the experiments and wrote the manuscript with input from co-authors. X.L. and J.W. performed all experiments. Q.Z., Yu Li, Yue Li, and K.L. helped animal studies. J.W., X.L., X.S., Q.W., and H.C. analyzed the data. E.D.A. provided *Glut1*^*f/f*^ mice and revised the manuscript. All authors discussed the results and commented on the manuscript.

## Supplemental Information

Document S1. Figures S1–S7 and Tables S1–S7

Document S2. Article plus Supplemental Information
