# Supplementary material for: Autophagy Reprograms Alveolar Progenitor Cell Metabolism in Response to Lung Injury
Source: Stem Cell Reports. 2020 Feb 13;14(3):420–32. doi: 10.1016/j.stemcr.2020.01.008 (PMC7066233; doi:10.1016/j.stemcr.2020.01.008)
Supplement: Document S1. Figures S1–S7 and Tables S1–S7 [file mmc1.pdf]

Stem Cell Reports, Volume *14*

Supplemental Information

Autophagy Reprograms Alveolar Progenitor Cell Metabolism in Response to Lung Injury

Xue Li, Junping Wu, Xin Sun, Qi Wu, Yue Li, Kuan Li, Qiuyang Zhang, Yu Li, E. Dale Abel, and Huaiyong Chen

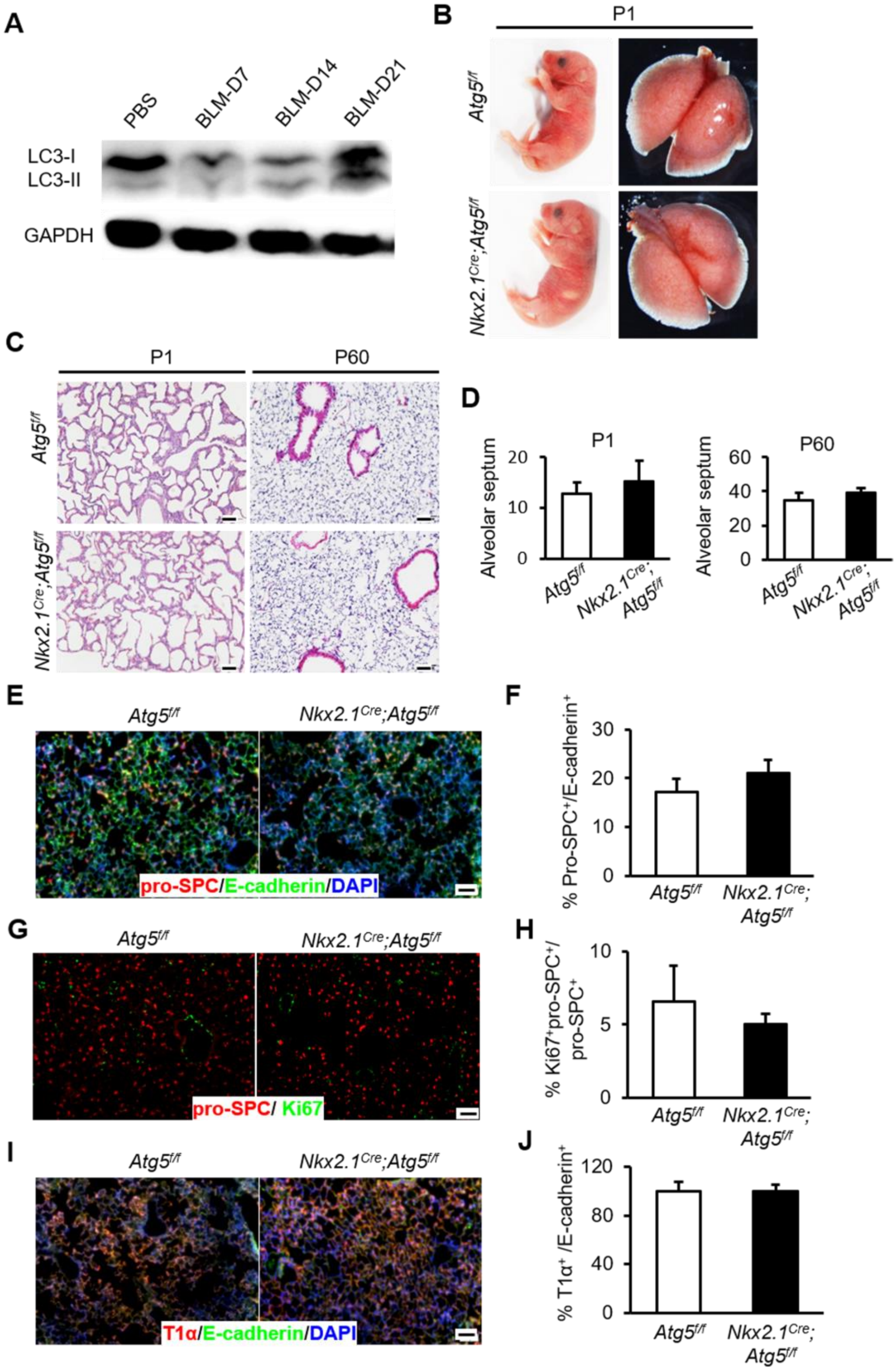

Figure S1. Autophagy in lung development and lung injury, related to Figure 1

(A) Representative images of western blot analysis of LC3-II and LC3-I expression in lung tissues harvested from mice at the indicated time points after bleomycin challenge (n=5). (B) Representative images of *Atg5*$^{f/f}$ mice and *Nkx2.1*^{Cre};*Atg5*$^{f/f}$ mice and their lungs at day 1 after birth (n=5). (C) Hematoxylin and eosin staining indicates no difference in lung sections from *Atg5*$^{f/f}$ mice and *Nkx2.1*^{Cre};*Atg5*$^{f/f}$ mice at day 1 and 60 after birth (n=5). (D) Alveolar septum is comparable between

Atg5$^{f/f}$ mice and *Nkx2.1*^{Cre};*Atg5*$^{f/f}$ mice (n=3). (E-F) IF staining demonstrates that pro-SPC^{+} cells in total E-cadherin^{+} cells is similar in lungs between adult *Atg5*$^{f/f}$ mice and *Nkx2.1*^{Cre};*Atg5*$^{f/f}$ mice (n=3). (G-H) No difference was observed in Ki67^{+}pro-SPC^{+} cells in total pro-SPC^{+} cells between adult *Atg5*$^{f/f}$ mice and *Nkx2.1*^{Cre};*Atg5*$^{f/f}$ mice (n=3). (I-J) Immunofluorescence staining demonstrates that the number of T1α^{+} cells in total E-cadherin^{+} cells is similar in lungs between adult *Atg5*$^{f/f}$ mice and *Nkx2.1*^{Cre};*Atg5*$^{f/f}$ mice (n=3). All data shown are mean ± SD. Scale bars, 50 μm.

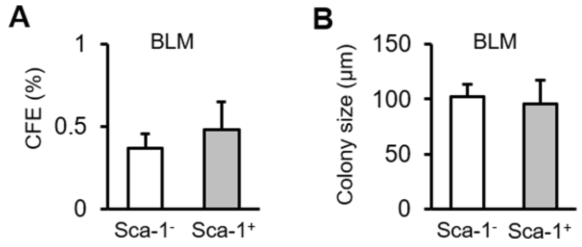

Figure S2. Sca1$^+$ alveolar epithelial cells and AT2 cells exhibit similar proliferation potential, related to Figure 1

(A-B) CFEs and Size of organoids derived from Sca-1$^+$ alveolar epithelial cells and AT2 cells isolated from mice 14 days after intratracheal instillation of bleomycin (n=3). All data shown are mean ± SD.

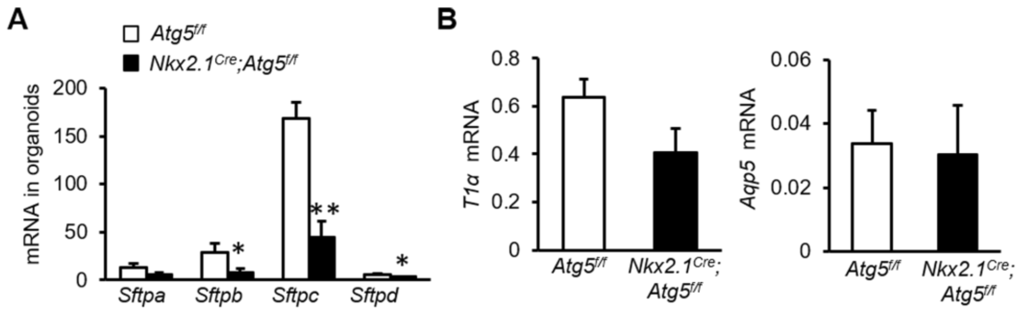

Figure S3. Expression of surfactant genes and AT1 marker genes in organoid cultures of survived AT2 cells during bleomycin injury, related to Figure 2

Quantitative PCR was conducted to analyze the expression of surfactant genes *Sftpa*, *Sftpb*, *Sftpc*, and *Sftpd* (A) and AT1 cell marker genes *T1α* and *Aqp5* (B) in organoid cultures of AT2 cells isolated from *Atg5$^{f/f}$* or *Nkx2.1^{Cre}*;*Atg5$^{f/f}$* mice at day 14 after BLM injury (n=4). *$p < 0.05$, **$p < 0.01$; all data shown are mean ± SD.

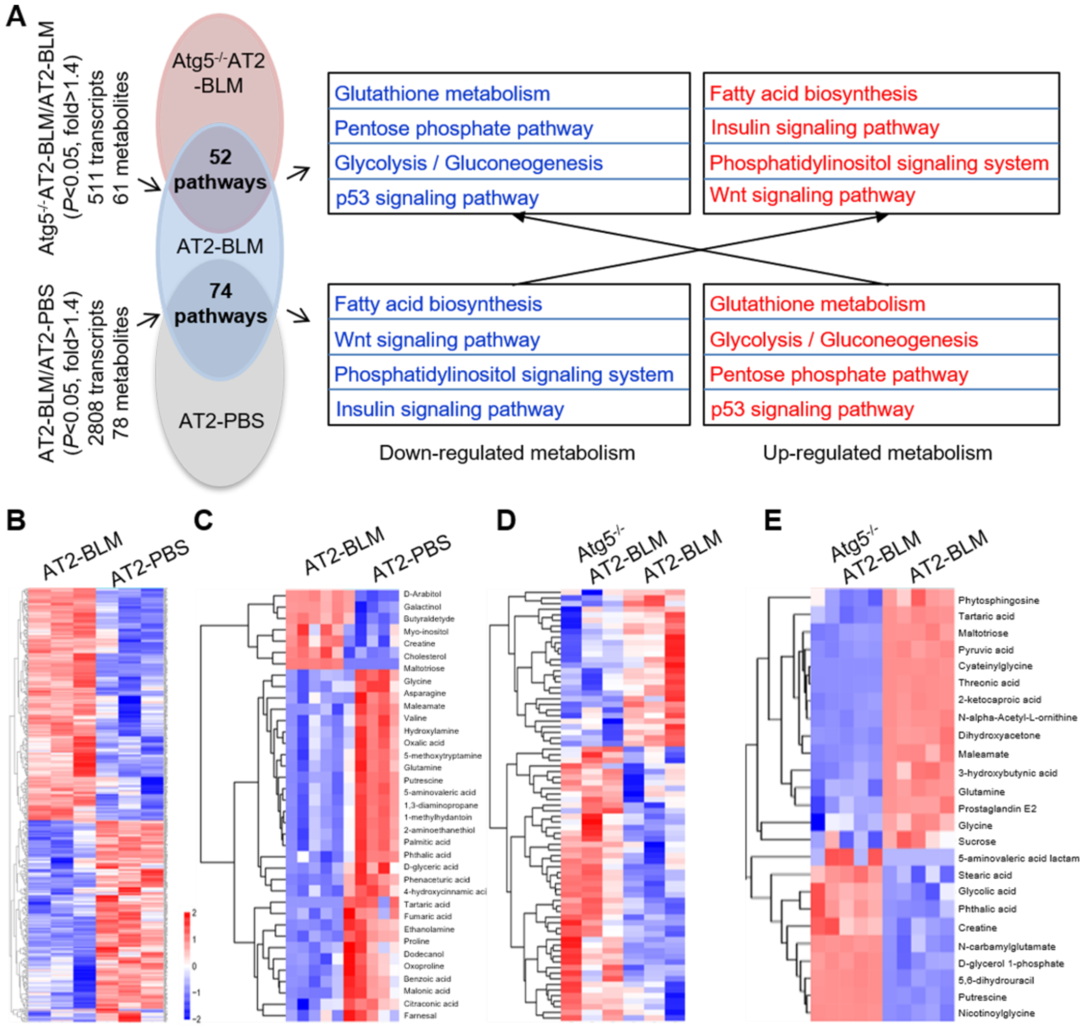

Figure S4. Autophagy drives metabolic changes in AT2 cells during bleomycin-induced lung injury, related to Figure 3

(A) Gene profiling showing that 2808 transcripts exhibited significant deregulation in AT2-BLM versus AT2-PBS (AT2-BLM / AT2-PBS, $p < 0.05$, >1.4-fold). Of the 188 detected metabolites, 78 were significantly altered. Integrated transcriptional and biochemical profiling identified 74 pathways altered in mouse AT2 cells from BLM- treated *Atg5*$^{f/f}$ mice (AT2-BLM) versus PBS-treated *Atg5*$^{f/f}$ mice (AT2-PBS). We observed 511 transcripts that were significantly deregulated in Atg5$^{-/-}$ AT2-BLM versus AT2-BLM (Atg5$^{-/-}$ AT2-BLM / AT2-BLM, $p < 0.05$, >1.4-fold). Sixty-one metabolites were significantly altered in Atg5$^{-/-}$ AT2-BLM versus AT2-BLM (Atg5$^{-/-}$ AT2-BLM / AT2-BLM, $p < 0.05$, >1.4-fold). Integrated transcriptomic/metabolic profiling of these significantly altered transcripts and metabolites revealed 52 deregulated metabolic pathways in AT2 cells in response to Atg5 loss during BLM- induced injury. (B) Heatmap showing transcriptional profiling of mouse AT2 cells isolated from control and BLM-challenged mice at day 14 (n=3). (C) Heatmap showing metabolic profiling of mouse AT2 cells isolated from control and BLM- challenged mice at day 14 (n=5). (D) Heatmap showing transcriptional profiling of mouse AT2 cells isolated from *Atg5*$^{f/f}$ and *Nkx2.1*^{Cre};*Atg5*$^{f/f}$ mice 14 d after BLM treatment (n=3). (E) Heatmap showing metabolic profiling of mouse AT2 cells isolated from *Atg5*$^{f/f}$ and *Nkx2.1*^{Cre};*Atg5*$^{f/f}$ mice 14 d after BLM treatment (n=5).

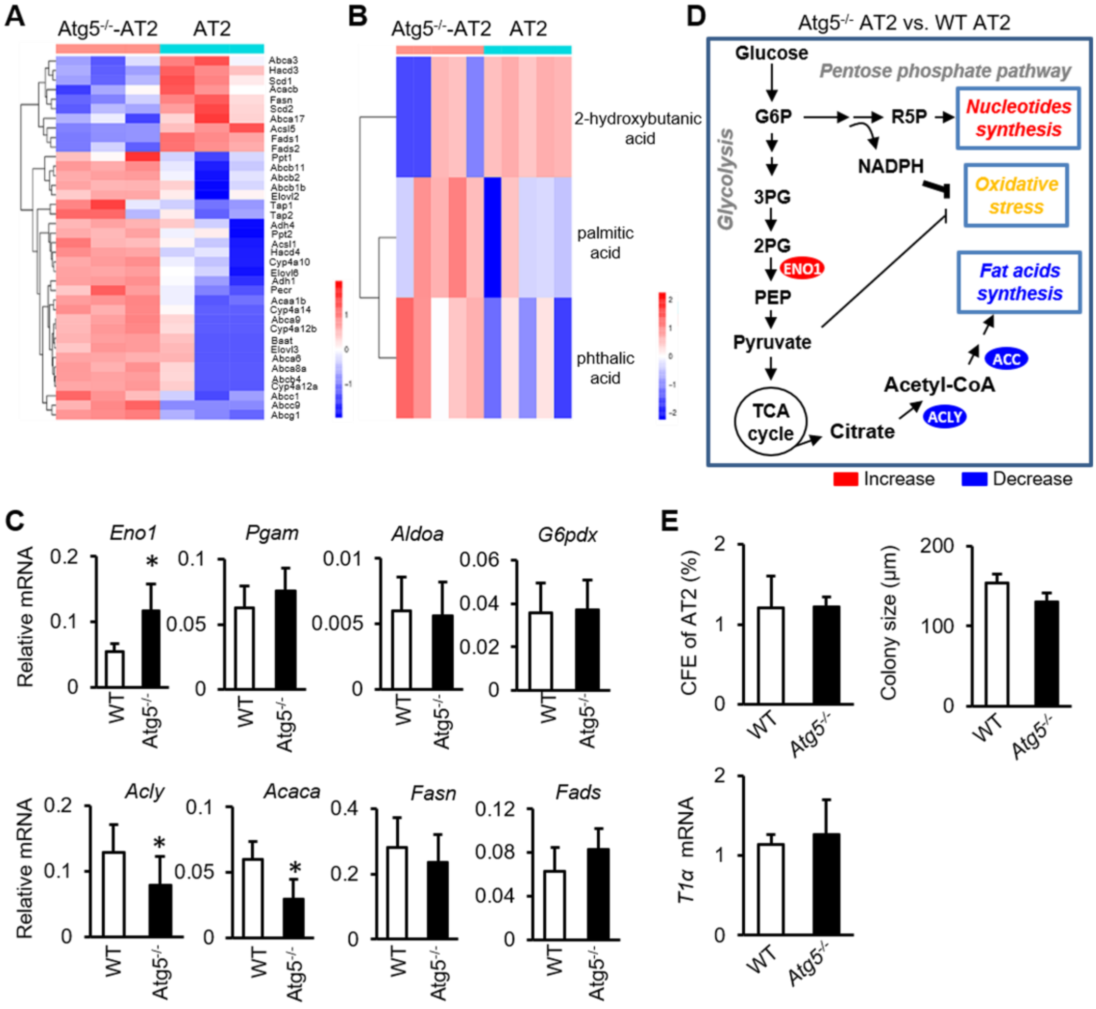

Figure S5. Role of autophagy in metabolism and function of mouse AT2 cells at steady-state, related to Figure 3

(A) Heatmap showing transcriptional profiling of mouse AT2 cells isolated from *Atg5*$^{f/f}$ (WT) control mice and *Nkx2.1*^{Cre}*; Atg5*$^{f/f}$ (*Atg5*$^{-/-}$) mice (n=3). (B) Heatmap of metabolic profiling of mouse AT2 cells isolated from WT and *Atg5*$^{-/-}$ mice (n=5). (C) qPCR validation of transcripts associated with glucose metabolism and fatty acid metabolism (n=3). (D) Schematic showing alterations in intermediates associated with glycolysis and synthesis of fatty acids. Increased and decreased intermediates are labeled in red and blue, respectively. Abbreviations: G6P, glucose 6-phosphate; 3PG, 3-phosphoglyceric acid; 2PG, 2-phosphoglyceric acid; and PEP, phosphoenolpyruvate. (E) CFEs, colony size and *T1α* expression in organoids of mouse AT2 cells isolated from WT and *Atg5*$^{-/-}$mice (n=5). $*p < 0.05$; all data shown are means ± SD.

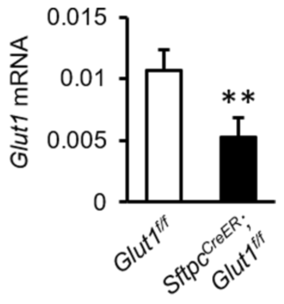

Figure S6. Validation of genetic deletion of Glut1 in AT2 cells from *Sftpc^{CreER};Glut1$^{f/f}$* mice, related to Figure 4

Quantitative PCR was conducted to analyze *Glut1* gene expression in AT2 cells isolated from *Glut1$^{f/f}$* mice and *Sftpc^{CreER};Glut1$^{f/f}$* mice (n=5). **p < 0.01; all data shown are mean ± SD.

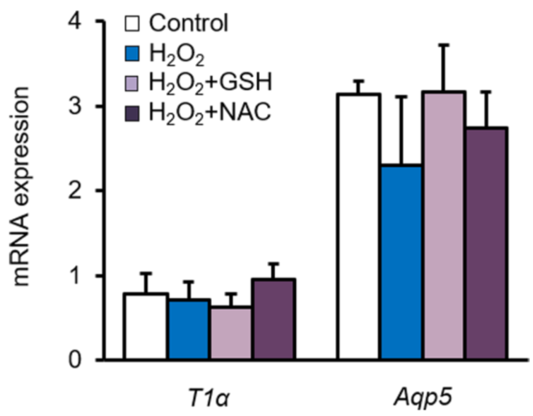

Figure S7. H_2O_2 does not regulate AT2 cell differentiation, related to Figure 7

Quantitative PCR was conducted to analyze the expression of *T1α* and *Aqp5* in organoid cultures of mouse AT2 cells under indicated conditions (n=6). All data shown are mean ± SD.

Table S1. Differential genes in AT2 cells in response to BLM injury, related to Figure 3

Gene	Ave. AT2-BLM	Ave. AT2-PBS	Fold AT2-BLM/AT2-PBS	*p*
Cxcl5	32.873	0.444	69.773	0.002
Lif	1.310	0.032	65.666	1.697E-11
Nkx2-9	39.206	0.479	51.586	8.914E-09
Fam46b	17.263	0.245	45.195	2.540E-05
Ly6a	583.417	12.983	39.776	1.466E-11
Tsku	1.878	0.035	36.678	4.577E-08
Psrc1	13.541	0.410	34.221	2.613E-17
Phlda3	151.932	4.336	32.347	7.375E-53
Mcam	7.832	0.211	31.491	8.707E-07
Cldn4	116.635	3.188	31.102	3.917E-04
Ifit1	6.439	0.238	26.075	6.949E-07
Upk1b	1.894	0.090	25.855	0.008
Klhl6	0.929	0.110	22.710	4.946E-04
Orm1	119.754	4.909	21.439	1.912E-36
Gsta2	4.843	0.216	20.501	3.272E-06
Rnf128	7.842	0.390	19.769	5.864E-21
Mthfd1l	0.403	0.046	19.323	0.015
Fblim1	2.915	0.201	18.354	9.933E-08
Wnt7a	3.210	0.137	17.337	0.034
Isg15	32.715	1.807	16.893	0.001
Tbx1	0.232	4.337	0.027	0.004
Gpr153	0.060	2.020	0.028	5.665E-05
Pon1	2.101	56.818	0.033	3.819E-10
Tmem132d	0.213	4.597	0.045	0.006
Cntfr	0.050	0.784	0.049	1.791E-04
Pde1b	0.532	7.799	0.055	4.605E-24
Cst8	5.318	77.543	0.059	4.551E-37
Cckar	0.903	13.161	0.068	1.292E-07
Bcl2a1a	1.766	14.770	0.069	5.380E-12
Smoc2	1.174	16.231	0.078	1.805E-17
Hes2	0.575	6.619	0.080	4.702E-06
Mturn	0.075	1.031	0.082	1.291E-05
Zmat4	0.144	1.232	0.082	3.265E-09
Avpr1a	0.889	9.322	0.086	2.979E-05
Bcan	1.006	10.118	0.090	1.234E-20
Tox3	0.051	0.380	0.090	0.003
Dll4	0.037	0.361	0.093	0.020
Fstl4	0.094	0.922	0.095	3.972E-04
Sclt1	0.248	1.557	0.095	3.107E-04
Vpreb3	3.365	27.085	0.096	5.653E-09

Table S2. Differential metabolites in AT2 cells in response to BLM injury, related to Figure 3

**Metabolites**	**Ave. AT2-BLM**	**Ave. AT2-PBS**	**Fold AT2-BLM/ AT2-PBS**	***p***
N-(2-hydroxyethyl)-iminodiacetic acid	5.937	0.681	8.715	5.840E-05
Galactinol	1152.647	236.396	4.876	0.003
Glucoheptonic acid	22.983	6.755	3.402	0.006
D-Arabitol	86.667	27.354	3.168	0.003
Erythrose	25.248	11.346	2.225	1.926E-04
Cholesterol	0.554	0.287	1.929	6.099E-05
Butyraldehyde	7.831	4.669	1.677	0.001
Oxamic acid	1.123	0.691	1.625	2.875E-05
3,6-Anhydro-D-galactose	0.548	0.368	1.489	0.017
Creatine	1.287	0.910	1.415	0.001
Itaconic acid	2.086	11.062	0.189	0.010
Dodecanol	1.745	7.579	0.230	0.037
4-Hydroxyquinazoline	0.201	0.847	0.237	0.003
Oxamide	0.603	2.289	0.264	0.007
Oxoproline	20.349	70.284	0.290	0.016
Fumaric acid	0.426	1.320	0.323	0.002
Citraconic acid	0.029	0.077	0.381	0.002
Pelargonic acid	0.328	0.806	0.407	7.404E-04
Asparagine	0.068	0.163	0.418	0.009
D-Altrose	1.391	3.295	0.422	3.048E-05

Table S3. Differential genes in Atg5$^{-/-}$ AT2 vs AT2 cells in response to BLM injury, related to Figure 3

Gene	Ave. Atg5$^{-/-}$ AT2-BLM	Ave. AT2-BLM	Fold Atg5$^{-/-}$ AT2-BLM/ AT2-BLM	*p*
Csf2rb2	0.392	0.082	9.001	0.031
Il27ra	0.338	0.041	8.938	0.023
Zfhx4	0.089	0.008	8.483	0.019
Nphs2	1.840	0.244	7.799	0.043
Cd200r4	0.420	0.077	6.960	0.030
Prkar2b	0.574	0.106	5.969	0.014
Aspn	0.348	0.107	5.843	0.042
Mturn	0.413	0.075	5.412	0.006
Ear2	15.719	5.016	5.324	0.016
Slc16a8	1.336	0.240	5.132	0.038
Gm14295	0.287	0.081	5.027	0.003
Rgs14	1.276	0.453	4.437	0.038
Gm14569	0.385	0.112	4.246	0.009
Cap1	11.631	2.611	4.005	3.267E-08
Tbc1d30	4.122	1.057	3.999	0.023
Phlpp1	0.720	0.267	3.973	0.008
Bcan	3.666	1.006	3.932	1.673E-05
Rfwd2	0.216	0.069	3.916	0.040
Prex1	0.900	0.304	3.891	0.004
Catsperg1	0.204	0.057	3.882	0.014
Serpinb9b	0.184	4.216	0.038	0.043
Fbln1	0.054	0.338	0.069	0.048
Reep2	0.060	0.547	0.091	0.049
Cmah	0.016	0.065	0.103	0.023
Zfp697	0.036	0.273	0.117	0.011
Anxa8	0.998	7.884	0.127	0.029
Cldn4	14.175	116.635	0.133	0.011
Gm13139	0.142	1.212	0.145	0.000
Lrtm2	0.186	1.071	0.176	0.023
Chdh	0.075	0.394	0.178	0.014
Gjb4	0.936	5.518	0.180	0.046
Slc4a11	0.423	2.322	0.195	0.027
Ptpdc1	0.151	0.907	0.202	0.002
Tdrkh	0.013	0.166	0.205	0.042
Ivl	1.874	10.087	0.207	0.006
Gm13251	0.062	0.332	0.210	0.019
Lif	0.303	1.310	0.219	0.000
Zdhhc15	0.162	0.722	0.221	0.017
Fam46b	3.508	17.263	0.222	0.020
Ugt1a6b	3.804	10.853	0.230	0.025

Table S4. Differential metabolites in Atg5$^{-/-}$ AT2 vs AT2 cells in response to BLM injury, related to Figure 3

**Metabolites**	**Ave. Atg5$^{-/-}$ AT2-BLM**	**Ave. AT2-BLM**	**Fold Atg5$^{-/-}$ AT2-BLM/ AT2-BLM**	***p***
Putrescine	38.162	0.185	206.070	4.933E-10
N-Methyl-DL-alanine	30.726	0.186	165.016	1.187E-08
2-Monopalmitin	14.283	0.109	130.588	5.613E-08
Nicotinoylglycine	25.328	0.199	127.117	5.905E-12
D-(glycerol 1-phosphate)	0.978	0.029	33.166	3.041E-07
Dibenzofuran	37.010	2.218	16.688	3.336E-12
Gallic acid	6.662	0.722	9.232	3.340E-12
Alpha-Ecdysone	1.131	0.201	5.631	1.088E-07
5,6-Dihydrouracil	305.699	57.348	5.331	9.025E-11
Glycolic acid	7.695	1.745	4.408	0.002
Threonic acid	0.104	33.969	0.003	6.027E-09
Halostachine	0.150	25.248	0.006	1.001E-06
Hydroxylamine	0.342	41.289	0.008	9.318E-10
2-Hydroxypyridine	1.961	161.315	0.012	3.330E-08
Pyruvic acid	0.194	14.890	0.013	1.525E-06
2-Ketocaproic acid	1.095	75.148	0.015	2.288E-08
N-Acetylisatin	0.414	14.816	0.028	3.428E-08
Aminooxyacetic acid	9.727	293.809	0.033	9.926E-12
Farnesal	3.110	86.667	0.036	9.336E-08
Maltotriose	0.227	5.738	0.040	2.129E-06

Table S5. Differential genes in Atg5$^{-/-}$ AT2 versus AT2 cells, related to Figure 3

Gene	Ave. Atg5$^{-/-}$ AT2	Ave. AT2	Fold Atg5$^{-/-}$ AT2/AT2	***p***
Sdc3	1.766	0.024	91.559	4.707E-15
Ms4a4c	3.887	0.048	91.116	1.067E-04
Ms4a6d	16.574	0.266	89.792	2.337E-23
Ifit1	18.466	0.238	85.797	0.003
Klra2	3.599	0.098	85.504	2.376E-24
Fcgr4	13.105	0.172	80.494	4.379E-23
Ifi204	8.553	0.124	75.610	0.002
Isg15	121.444	1.807	70.553	7.611E-04
Cd22	1.711	0.018	66.423	1.536E-04
Sla	8.707	0.087	65.336	2.464E-19
Ear2	206.779	4.843	63.104	1.491E-93
Ccr1	8.709	0.136	61.330	1.308E-25
Cd84	1.600	0.031	59.509	1.636E-25
Ifit3	21.963	0.430	58.318	0.004
Arhgap30	3.561	0.062	57.993	5.231E-23
Nkx2-9	36.963	0.479	56.612	5.666E-30
Dkk3	1.345	0.050	54.022	1.533E-06
Chil3	732.883	13.674	52.479	2.364E-32
Apbb1ip	1.465	0.040	51.369	2.765E-08
Wdfy4	0.673	0.009	51.067	1.066E-10
Ddx3y	0.141	2.984	0.044	0.007
Eif2s3y	0.409	5.785	0.068	0.015
LOC108168906	0.006	0.056	0.074	0.032
Zfp618	0.080	0.578	0.113	6.356E-06
Frat2	0.473	3.248	0.119	2.214E-04
BC021891	0.040	0.284	0.126	0.034
Lca5l	0.093	0.436	0.134	0.005
Fstl4	0.154	0.922	0.148	0.002
Tmem132c	0.248	1.271	0.153	7.512E-05
Avpr1a	1.511	9.322	0.162	5.659E-04
Gpr153	0.428	2.020	0.168	0.007
Tdg	0.783	4.439	0.169	0.002
Nipa1	1.081	7.180	0.170	0.015
Pcdhga12	0.146	0.903	0.205	0.042
Cecr6	0.214	1.240	0.205	7.623E-04
Tmem238	6.086	28.856	0.205	0.025
Mtmr11	0.804	4.027	0.213	1.700E-06
Scarf2	6.273	25.091	0.214	0.021
Adamts1	3.920	16.955	0.218	7.902E-08
Ppp1r1a	2.695	11.402	0.218	9.969E-06

Table S6. Differential metabolites in Atg5$^{-/-}$ AT2 versus AT2 cells, related to Figure 3

**Metabolites**	**Ave. Atg5$^{-/-}$ AT2**	**Ave. AT2**	**Fold Atg5$^{-/-}$ AT2/AT2**	***p***
Levoglucosan	1.961	1.386	1.415	0.048
Phthalic acid	2.197	1.839	1.195	0.015
Palmitic acid	25.822	21.831	1.183	0.045
Alanine	22.108	20.449	1.081	0.022
2-Hydroxybutanoic acid	0.123	0.362	0.340	0.034

Table S7. Sequences of primers for quantitative PCR

Gene	Forward primer	Reverse primer
β-actin	5’-GGCCAACCGTGAAAAGATGA-3’	5’-CAGCCTGGATGGCTACGTACA-3’
E-cadherin	5’-CTGCTGCTCCTACTGTTTCTAC-3’	5’-TCTTCTTCTCCACCTCCTTCT-3’
T1α	5’-TGCTACTGGAGGGCTTAATGA-3’	5’-TGCTGAGGTGGACAGTTCCT-3’
Aqp5	5’-GGTGGTCATGAATCGGTTCAGC-3’	5’-GTCCTCCTCTGGCTCATATGTG-3’
Sftpa	5’-CATCAGATTCTGCAAACAATGGG-3’	5’-GGCTCTGGTACACATCTCTCTAA-3’
Sftpb	5’-ACAAGGCCCTCAATTCTGGTGC-3’	5’-CAGGTCATTAGCTCCTGCATGC-3’
Sftpc	5’-GAAGATGGCTCCAGAGAGCATC-3’	5’-GGACTCGGAACCAGTATCATGC-3’
Sftpd	5’-CCTCTCGCAGAGATCAGTACC-3’	5’-CCATCCCGTCCATCACGAC-3’
Atg5	5’-TGAAAGAGAAGCAGAACCATACT-3’	5’-GGGTGTGCCTTCATATTCAAAC-3’
G6pdx	5’-CCACTCCAGAAGAAAGACCTAAG-3’	5’-TGGCTGTTGAGGTGCTTATAG-3’
Pgam	5’-GGTCTGACAGGTCTCAACAAA-3’	5’-GGCGGTGGGACATCATAAG-3’
Eno1	5’-GATGGACGGCACAGAGAATAAA-3’	5’-TCAGCAATGTGGCGGTAAA-3’
Aldoa	5’-CCCAGCAACAGACAGAGTTAG-3’	5’-GATGTCAGACAGCTCCTTCTTC-3’
Acly	5’-CTCACACGGAAGCTCATCAA-3’	5’-TCCAGCATTCCACCAGTATTC-3’
Fasn	5’-AGACCCGAACTCCAAGTTATTC-3’	5’-GCAGCTCCTTGTATACTTCTCC-3’
Fads	5’-GCTTTGAACCCACCAAGAATAAA-3’	5’-CAGGATGTGAAGCAGGTAGAC-3’
Acaca	5’-ACATTCCGAGCAAGGGATAAG-3’	5’-GGGATGGCAGTAAGGTCAAA-3’
Glut1	5’-CCTCGTGCTCTTCTTCATCTT-3’	5’-CTCGGGTGTCTTGTCACTTT-3’

Key resources table

**Reagents or Resources**	**Source**	**Identifier**
Antibodies		
CD24-PE	eBioscience	Cat #: 12-0242-81
EpCAM-PE-Cyanine7	eBioscience	Cat #: 25-5791-80
Sca1-APC	eBioscience	Cat #: 17-5981-81
CD31 Biotin	eBioscience	Cat #: 13-0311-85
CD34 Biotin	eBioscience	Cat #: 13-0341-85
CD45 Biotin	eBioscience	Cat #: 13-0451-85
Streptavidin APC-eFluor 780	eBioscience	Cat #: 47-4317-82
7-AAD	eBioscience	Cat #: 00-6993-50
DAPI Fluoromount G	Southern Biotech	Cat #: 0100-20
anti-Mouse Alexa Fluor 488	Invitrogen	Cat #: A21202
anti-Rabbit Alexa Fluor 594	Invitrogen	Cat #: A21207
anti-Rat Alexa Fluor 488	Invitrogen	Cat #: A21208
anti-Hamster Alexa Fluor 488	Invitrogen	Cat #: A21110
E-cadherin (mouse)	BD	Cat #: 610181
T1α (hamster)	eBioscience	Cat #: 14-5381-81
pro-SPC (rabbit)	Millipore	Cat #: ab3786
Ki67 (rat)	eBioscience	Cat #: 14-5698-82
LC3 (rabbit)	Sigma	Cat #: L7543
GAPDH	Sungene Biotech	Cat #: KM9002S
Anti-Rabbit IgG (H+L)	Thermo Fisher	Cat #: 31460
Chemicals, Peptides, and Recombinant Proteins		
O.C.T. compound	Tissue-Tek	Cat #: 4583
Hydrogen peroxide solution	Sigma	Cat #: H1009-5mL
L-Glutathione reduced (GSH)	Sigma	Cat #: V900456-5G
N-acetyl-L-cysteine (NAC)	Sigma	Cat #: A7250-10G
2-Deoxyglucose (2-DG)	Sigma	Cat #: D8375-1G
6-amino-nicotinamide (6-AN)	Sigma	Cat #: A68203-1G
3-Bromopyruvic (3-BrPA)	Santa Cruz	Cat #: sc-260854
BMS-303141	Tocris Bioscience	Cat #: 4609
Orlistat	Sigma	Cat #: O4139-25mg
Tamoxifen	Sigma	Cat #: T5648-1G
Corn Oil	Sigma	Cat #: C8267-500mL
Elastase	Worthington	Cat #: LS002279

	Biochemical Corporation	
Growth factor reduced Matrigel	BD Pharmingen	Cat #: 356231
Masson's trichrome stain	MXB Biotechnologies	Cat #: MST-8004
Hematoxylin	ZSQB-BIO	Cat #: 322350
Eosin solution	ZSQB-BIO	Cat #: ZLI9613
Fluorometric Hydrogen Peroxide Assay Kit	Sigma	Cat #: MAK165-1KT
Insulin/transferrin/selenium	Sigma	Cat #: I3146
DMEM/F12	Gibco	Cat #: 11320-033
Ethylene glycol-bis (EGTA)	Sigma	Cat #: E8145-10G
Deoxyribonuclease 1	Sigma	Cat #: DN-25
SB431542	Sigma	Cat #: S4317
Penicillin-streptomycin	Gibco	Cat #: 15140-122
HEPES	Sigma	Cat #: H0087
Thincert cell culture insert	Greiner Bio-One	Cat #: 662641
Hank's Balanced Salt Solution (HBSS)	Cellgro	Cat #: 21-023-CV
FBS	Gibco	Cat #: 16000-044
Red Blood Cell Lysing Buffer	Sigma	Cat #: R7767-100mL
TRIzol™ LS Reagent	Invitrogen	Cat #: 10296010
miRNA Isolation Kit	Ambion	Cat #: 1561
TruSeq Stranded mRNA LTSample Prep Kit	Illumina	Cat #: RS-122-2101
Agencourt AMPure XP	BECKMAN COULTER	Cat #: A63881
SSIII	Invitrogen	Cat #: 18080044
MLV	Invitrogen	Cat #: S28025-014
RRI	TaKaRa	Cat #: 2313A
Radom Primers	TaKaRa	Cat #: D3801
dA/T/C/GTP	TaKaRa	Cat #: D4026A/D4029A/ D4028A/D4027A
10xPCR Buffer	Roche	Cat #: 11699105001
$MgCl_2$	Roche	Cat #: 11699113001
SYBR SELECT MASTER MIX	Applied Biosystems	Cat #: 4472908
Experimental Models: Organisms/Strains		
Sftpc-CreER™	Jackson Lab.	Stock #: 028054
Nkx2.1-Cre	Jackson Laboratories	Stock #: 008661

Atg5$^{f/f}$	The RIKEN BRC	RBRC02975
Glut1$^{f/f}$	N/A	N/A
Biological Samples: Cell Lines		
MLg2908	ATCC	CCL-206
Software and Algorithms		
Trimmomatic	v0.36	LEADING:3 TRAILING:3 SLIDINGWINDOW:4:15 MINLEN:50
Cufflinks	v2.2.1	library-type fr-firststrand
DESeq	v1.18.0	pvalue<0.05, |log2FoldChange|>1
Pheatmap	v3.2.0	R package
